# Supplementary material for: RIPOR2 promotes multinucleation of melanoma cells downstream of the RAS/ERK oncogenic pathway
Source: iScience. 2026 Apr 15;29(5):115734. doi: 10.1016/j.isci.2026.115734 (PMC13145898; doi:10.1016/j.isci.2026.115734)
Supplement: Document S1. Figures S1–S19 [file mmc1.pdf]

iScience, Volume *29*

Supplemental information

RIPOR2 promotes multinucleation of melanoma cells downstream of the RAS/ERK oncogenic pathway

Axelle Wilmerding, Aurélie Richard, Nicolas Macagno, Estelle Hirsinger, Tarek Gharsalli, Léa Bellenger, Naïra Naouar, Caroline Gaudy, Stéphanie Mallet, Nathalie Degardin, Lauranne Bouteille, Nathalie Caruso, Delphine Duprez, Yacine Graba, Souhila Medjkane, Heather C. Etchevers, and Marie-Claire Delfini

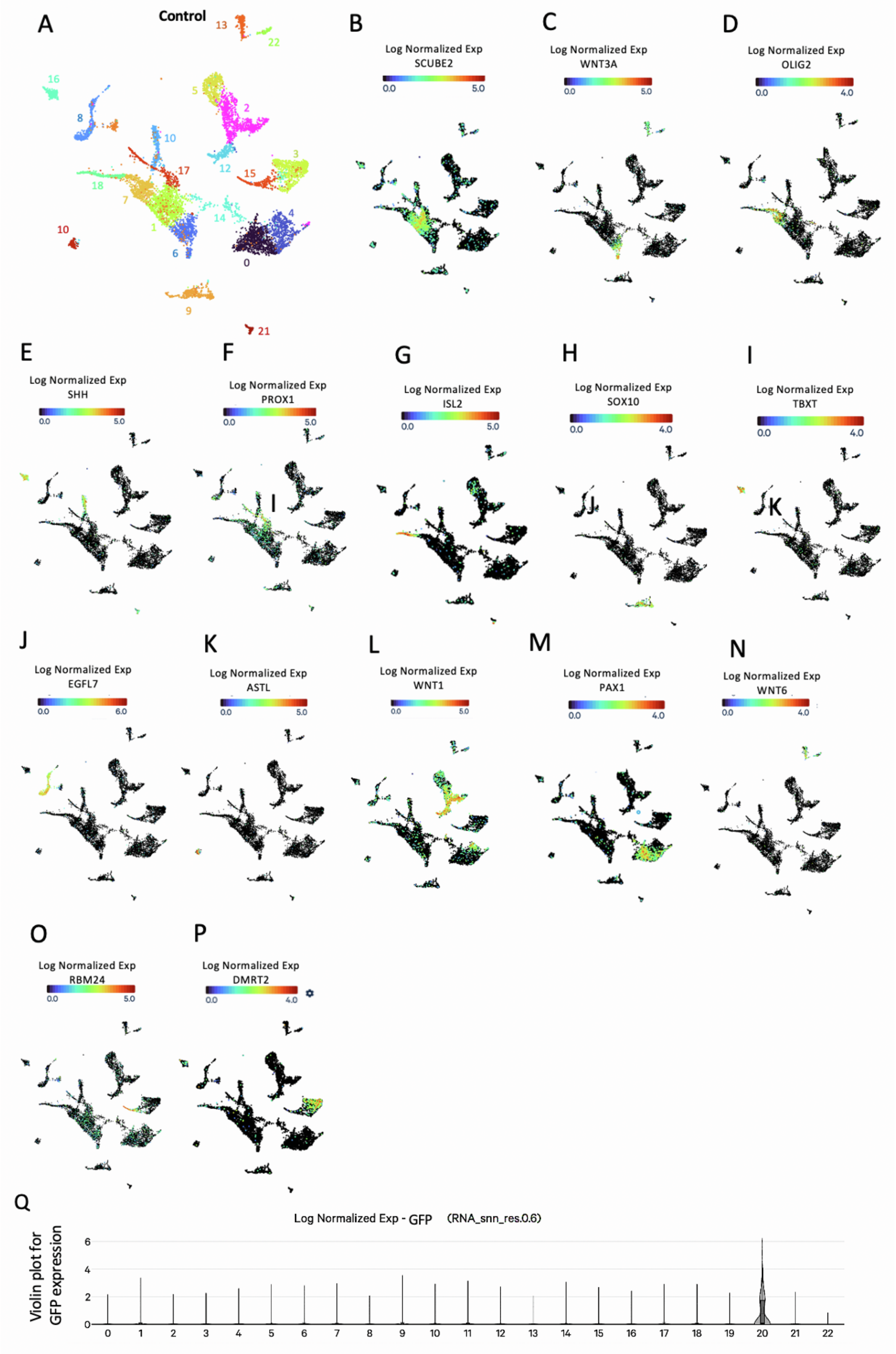
A
Control
B
Log Normalized Exp
SCUBE2
C
Log Normalized Exp
WNT3A
D
Log Normalized Exp
OLIG2
E
Log Normalized Exp
SHH
F
Log Normalized Exp
PROX1
G
Log Normalized Exp
ISL2
H
Log Normalized Exp
SOX10
I
Log Normalized Exp
TBXT
J
Log Normalized Exp
EGFL7
K
Log Normalized Exp
ASTL
L
Log Normalized Exp
WNT1
M
Log Normalized Exp
PAX1
N
Log Normalized Exp
WNT6
O
Log Normalized Exp
RBM24
P
Log Normalized Exp
DMRT2
Q
Log Normalized Exp - GFP (RNA_snn_res.0.6)
Violin plot for GFP expression

FIG SUP 1

Supplementary Figure 1: UMAP, Feature and Violon plots obtained from the single-nucleus RNA sequencing for the control condition.

A - UMAP plot at snn_res.0.6 for the control condition (pCIG nuclei only). Feature plot of the control condition nuclei only for the *SCUBE2* gene (B), the *WNT3A* gene (C), the *OLIG2* gene (D), the *SHH* gene (E), the *PROX1* gene (F), the *ISL2* gene (G), the *SOX10* gene (H), the *TBXT* gene (I), the *GHFL7* gene (J), the *ASTL* gene (K), the *WNT1* gene (L), the *PAX1* gene (M), the *WNT6* gene (N), the *RBM24* gene (O), and the *DMRT2* gene (P). Q- Violin plots for GFP expression showing the distribution of transfected cells in each cluster.

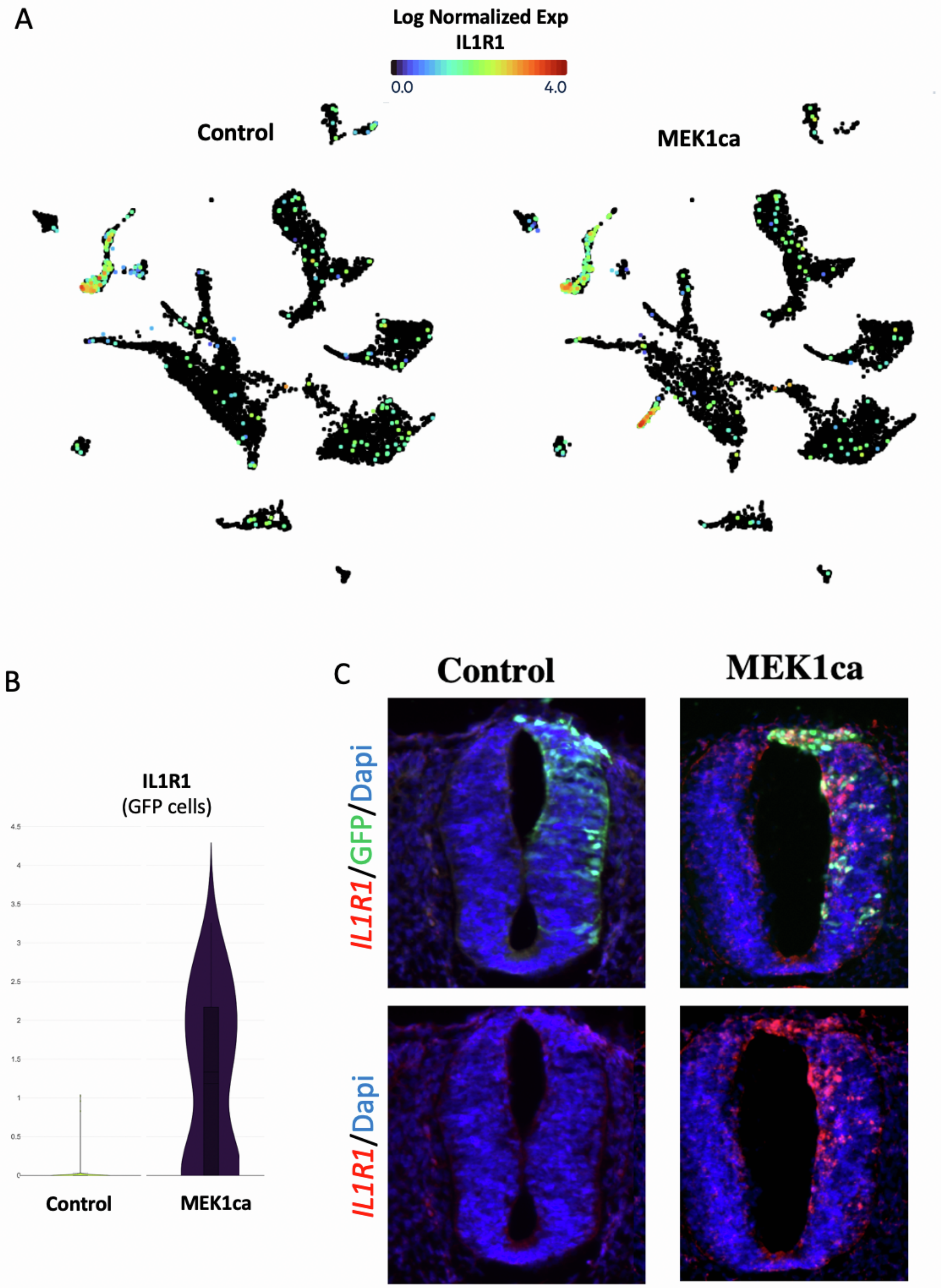
A
Log Normalized Exp
IL1R1
0.0
4.0
Control
MEK1ca
B
IL1R1
(GFP cells)
4.5
4
3.5
3
2.5
2
1.5
1
0.5
0
Control
MEK1ca
C
Control
MEK1ca
IL1R1/GFP/Dapi
IL1R1/Dapi

FIG SUP 2

Supplementary Figure 2: *IL1R1* expression is upregulated after MEK1ca expression in the neural tube.

A - Feature plot of the two conditions (control and MEK1ca) for the *IL1R1* gene. **B -** Violin plot of *IL1R1* in transfected nuclei (GFP > 1). **C -** Fluorescence in situ hybridization with the *IL1R1* probe and immunofluorescence staining with the anti-GFP antibody on transverse trunk sections of chicken embryos one day after electroporation in the control (pCIG) or MEK1ca conditions.

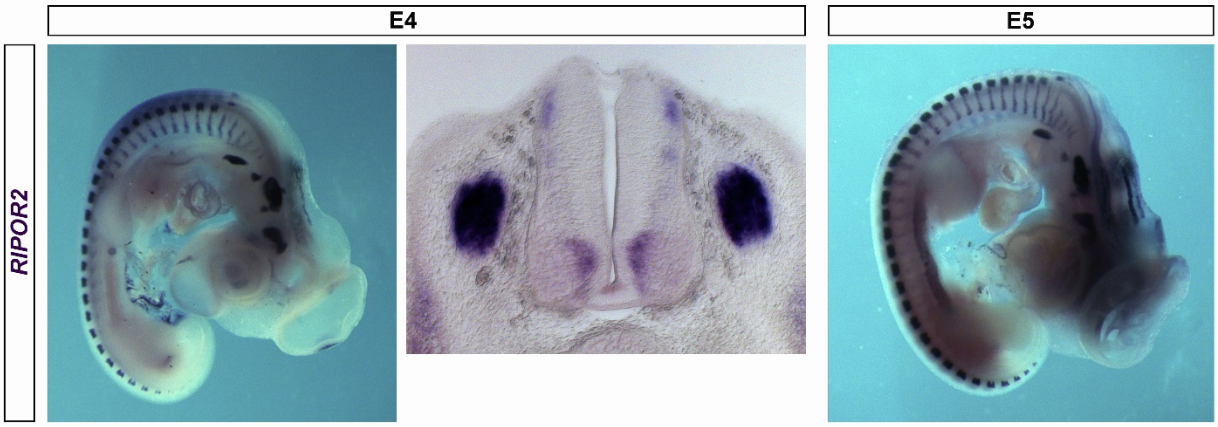

Supplementary Figure 3: *RIPOR2* expression in chicken embryo.

Lateral view and transverse section of E4 chicken embryo and lateral view of E5 chicken embryo after whole-mount in situ hybridization with the chicken *RIPOR2* probe.

A

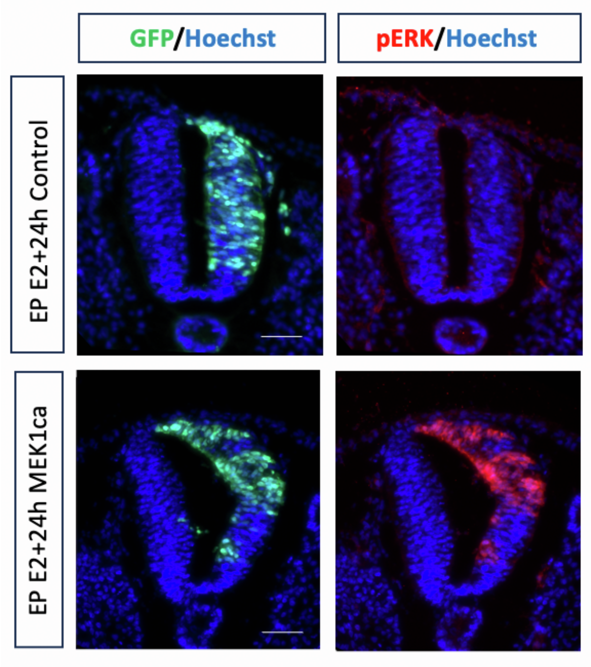

B

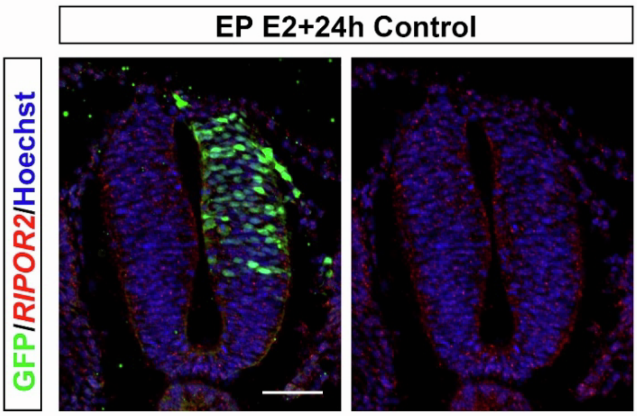

Supplementary Figure 4: MEK1ca expression in the neural tube of chicken embryo leads to an overactivation of ERK1/2.

A - Immunofluorescence staining on trunk transverse sections of chicken embryo with anti-GFP and anti-pERK1/2 antibodies one day post-electroporation with the control plasmid (pCIG) and with the pCIG-MEK1ca-expressing vectors. **B -** Fluorescent *in situ* hybridization with a chicken *RIPOR2* probe and immunofluorescence staining with the anti-GFP antibody on trunk transverse section of chicken embryo one day after electroporation of the control plasmid (pCIG). Blue represents Hoechst nuclear staining. Scale bar: 50 µm.

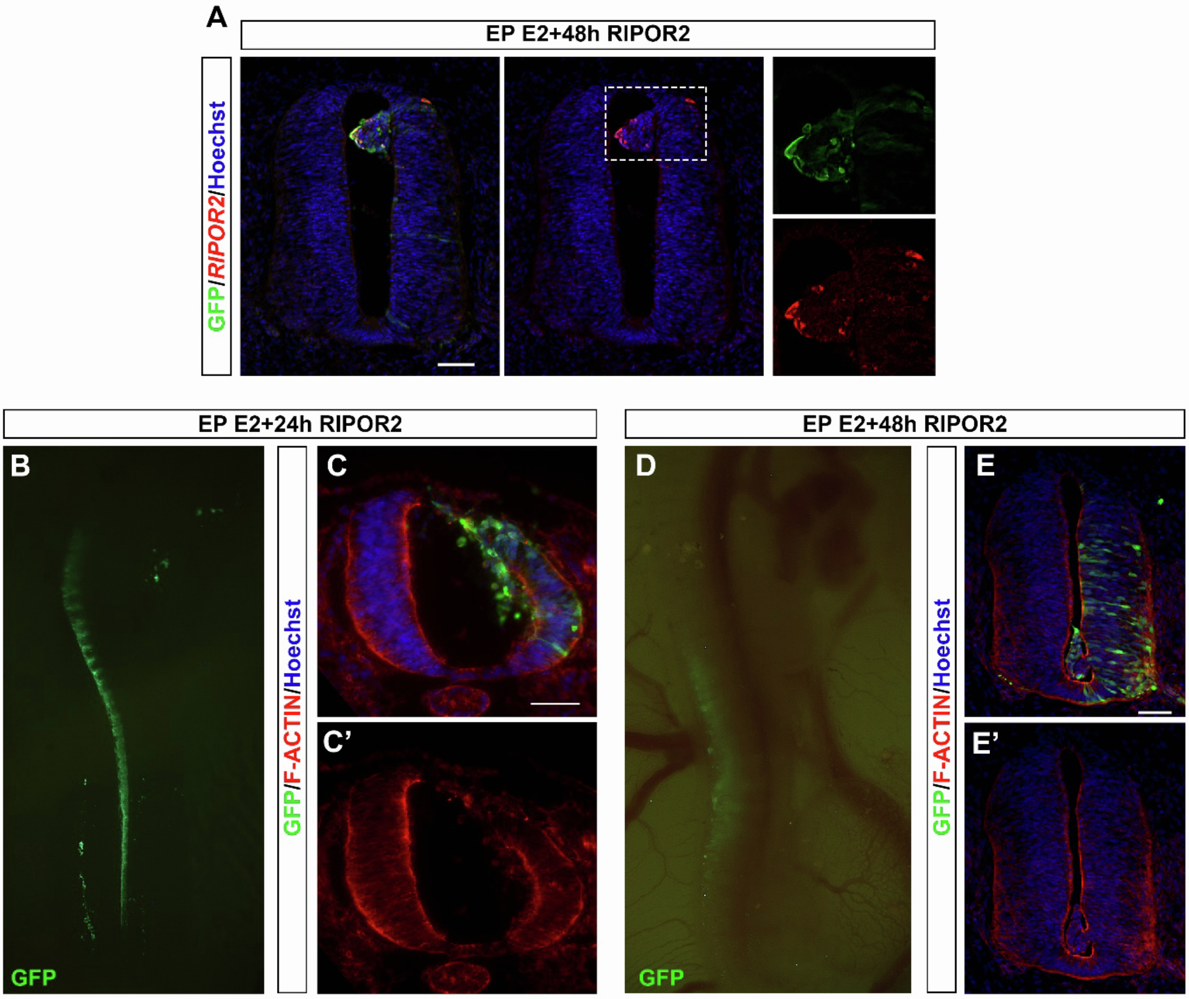

Supplementary Figure 5: The gain-of-function of RIPOR2 in the trunk neural tube in chicken embryos disrupts the neuroepithelium.

A - Fluorescent *in situ* hybridization with a chicken RIPOR2 probe and immunofluorescence with the anti-GFP antibody on trunk transverse section of chicken embryo two days after electroporation of RIPOR2. **B -** Dorsal view of a whole-mount embryo one day after the electroporation of the RIPOR2-expressing vector under fluorescent binocular highlighting GFP+ transfected cells (left side). **C-C' -** Immunofluorescence staining with the anti-GFP antibody and F-ACTIN staining on trunk transverse section of chicken embryo one day after electroporation of RIPOR2, highlighting the disorganization of the neuroepithelium with cells invading the lumen of the neural tube. **D -** Dorsal view of a whole-mount embryo two days after the electroporation of the RIPOR2-expressing vector using a fluorescent binocular microscope. **D-D' -** Immunofluorescence with the anti-GFP antibody and F-ACTIN staining on trunk transverse section of chicken embryo two days after electroporation of RIPOR2. Blue represents Hoechst nuclear staining. Scale bar: 50 µm.

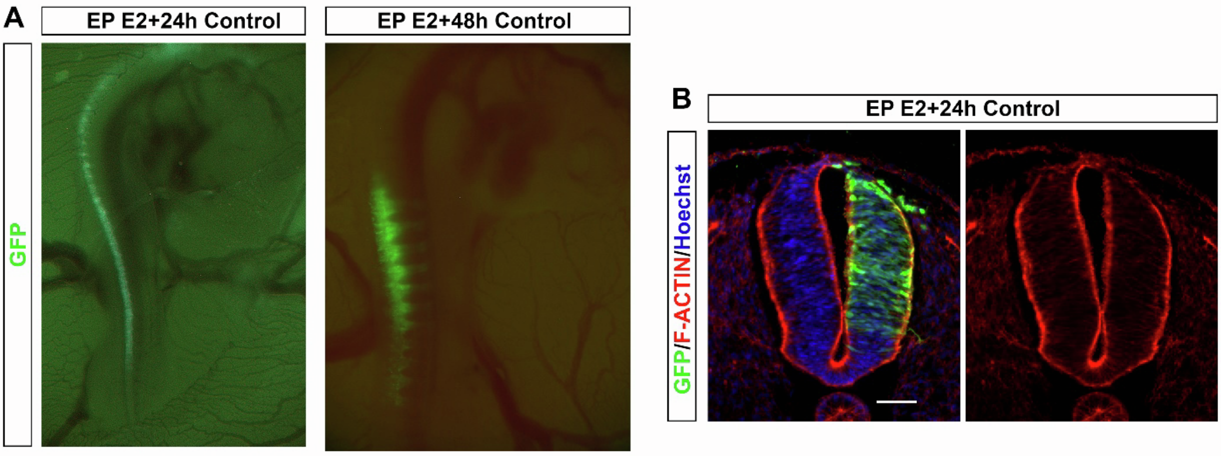

Supplementary Figure 6: Expression of control plasmid in the chicken neural tube.

A - Dorsal view of whole-mount embryo one day and two days after the electroporation of the control plasmid (pCAGGS, expressing only GFP) using a fluorescent binocular microscope, highlighting GFP+ transfected cells. **B -** Immunofluorescence staining with the anti-GFP antibody and F-ACTIN staining on trunk transverse section of chicken embryo one day after electroporation of the control plasmid. Blue represents Hoechst nuclear staining. Scale bar: 50 µm.

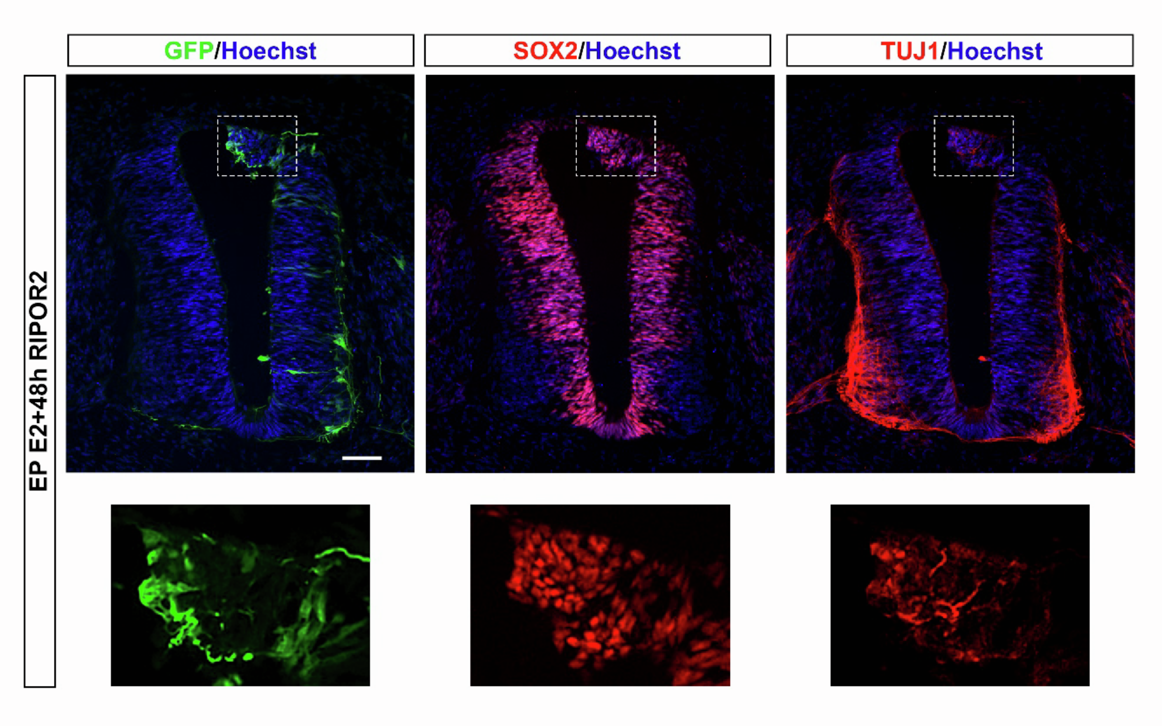

Supplementary Figure 7: Immunostraining against the progenitor marker SOX2 and the pan-neuronal marker TUJ1 reveals neuroepithelial disorganization induced by RIPOR2 electroporation.

Immunofluorescence staining with the anti-GFP, anti-SOX2, and anti-TUJ1 antibodies on trunk transverse sections of chicken embryo two days after electroporation of RIPOR2, highlighting the disorganization of the neuroepithelium as TUJ1 staining is observed at the apical part of the neural tube. Dotted boxes are magnified in the bottom panel. Blue represents Hoechst nuclear staining. Scale bar: 50 µm.

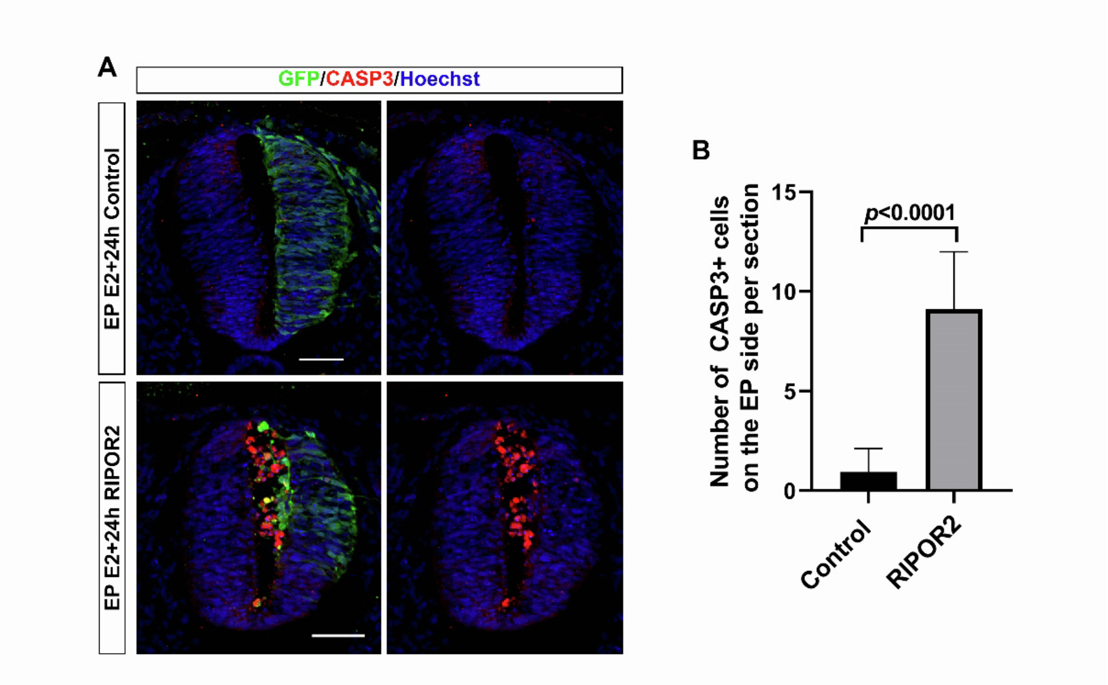

Supplementary Figure 8: Immunofluorescence staining with CASP3 antibody reveals that RIPOR2 gain-of-function in the trunk neural tube triggers massive apoptosis.

A - Immunofluorescence staining with the anti-GFP and anti-CASP3 antibodies on trunk transverse sections of chicken embryos one day after electroporation of the control vector (pCAGGS) and RIPOR2 vector. Blue represents Hoechst nuclear staining. Scale bar: 50 µm. **B -** Quantification of the number of CASP3+ cells on the electroporated side between the control (pCAGGS) and RIPOR2 conditions (n=3 animals, 18 sections, two-tailed Mann–Whitney test, error bars represent s.d.).

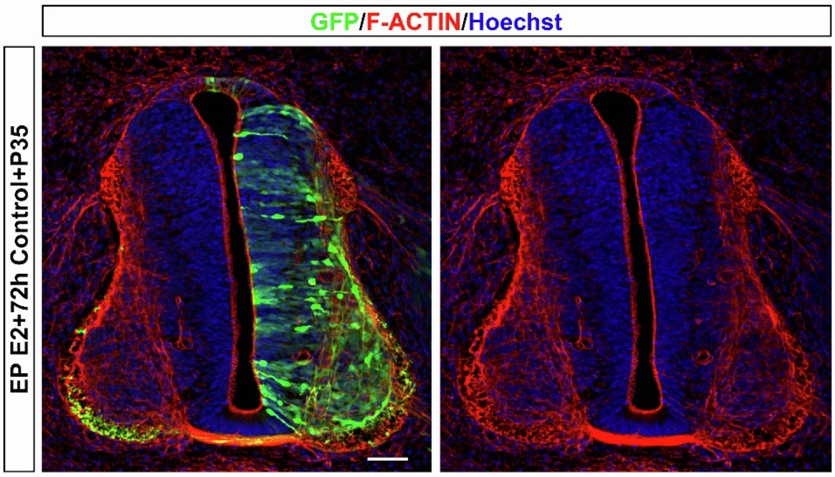

Supplementary Figure 9: F-ACTIN staining after expression of control and P35 plasmids in the chicken neural tube.

Immunofluorescence staining with the anti-GFP antibody and F-ACTIN staining on trunk transverse section of a chicken embryo three days after electroporation of the control vector (pCAGGS) and P35 vector. Blue represents Hoechst nuclear staining. Scale bar: 50 µm.

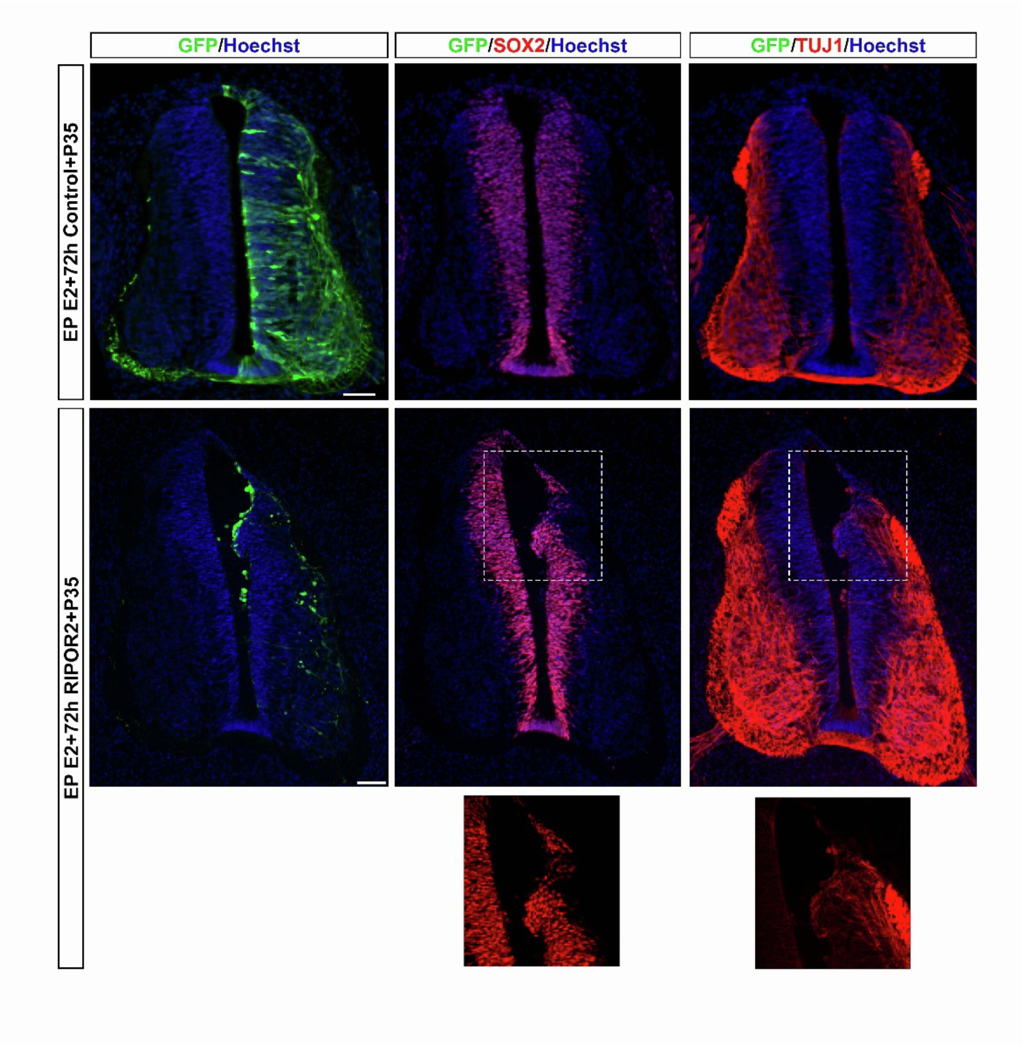

Supplementary Figure 10: SOX2 and TUJ staining after expression of control and P35 plasmids in the chicken neural tube.

Immunofluorescence staining with the anti-GFP, anti-SOX2, and anti-TUJ1 antibodies on trunk transverse sections of a chicken embryo three days after electroporation of the control (P35 vector only) or RIPOR2 andP35 vectors. Reduced Sox2 and increased TUJ1 staining can be observed at the apical part of the neural tube in the RIPOR2 and P35 condition. Dotted boxes are magnified in the bottom panel. Blue represents Hoechst staining. Scale bar: 50 µm.

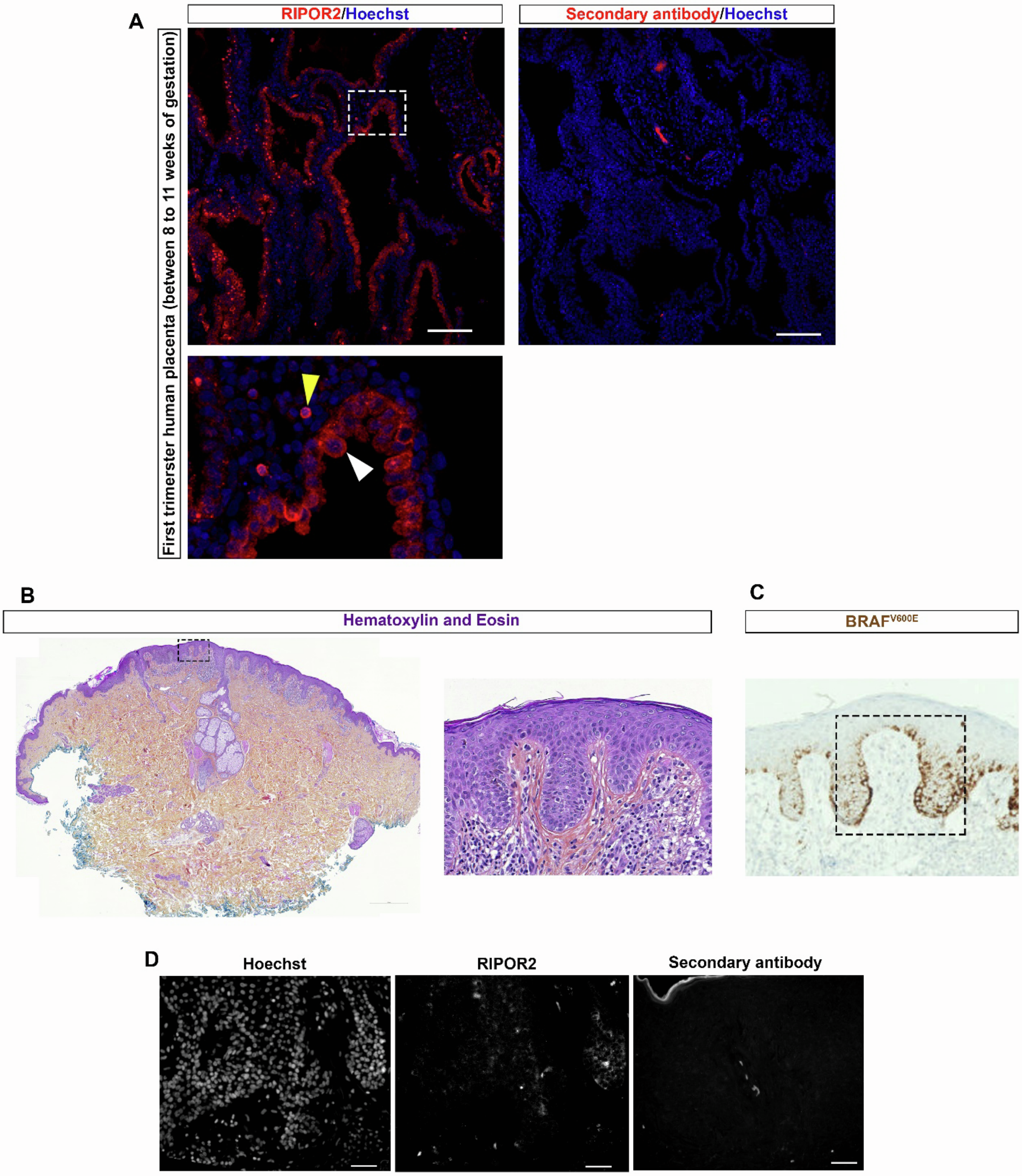
A
RIPOR2/Hoechst
Secondary antibody/Hoechst
First trimerster human placenta (between 8 to 11 weeks of gestation)
B
Hematoxylin and Eosin
C
BRAFV600E
D
Hoechst
RIPOR2
Secondary antibody

FIG SUP 11

Supplementary Figure 11: Validation of RIPOR2 antibody.

A- Immunofluorescence staining was performed with the RIPOR2 antibody on transverse section of human placenta from the first trimester (8-11 weeks of gestation). RIPOR2 is expressed at the outer edge of the syncytiotrophoblast (white arrowhead), as previously described (Darkour et al., 1997). Note that RIPOR2 is also expressed in immune cells (yellow arrowhead). The secondary antibody alone (rabbit-647) was used as a control. Blue corresponds to Hoechst nuclear staining in all panels. **B-D-** Adjacent sections of a $BRAF^{V600E}$-benign melanocytic nevi. Dotted boxes are magnified in **D**. **B-** H&E stain shows tissue disorganization. **C-**Immunohistochemistry with an anti-$BRAF^{V600E}$ antibody stains the mutated melanocytes and highlights the malignant lesion zone. **D-** Hoechst nuclear staining and anti-RIPOR2 antibodies staining. The secondary antibody alone (rabbit-647) was used as a control. Scale bar: 100µm.

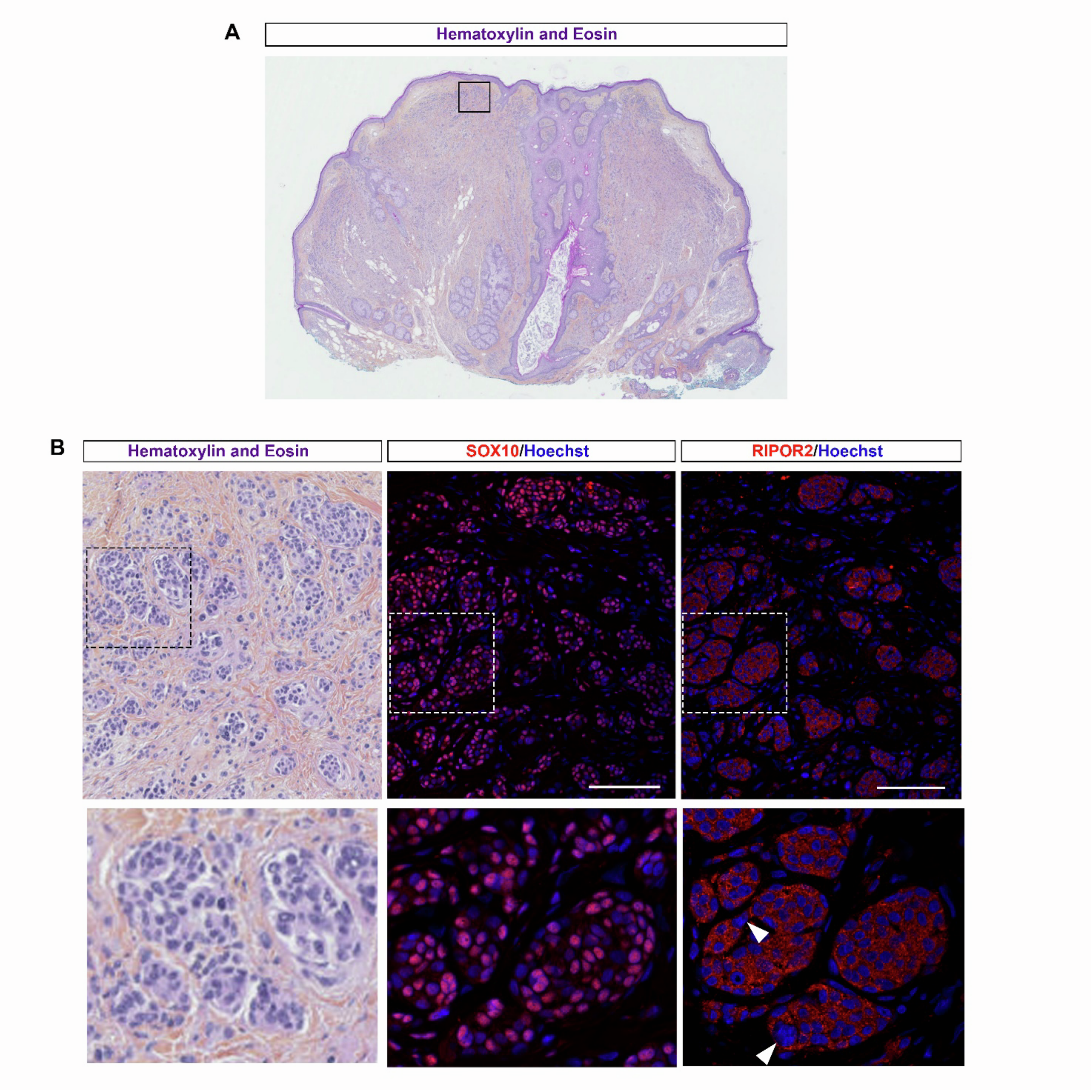

Supplementary Figure 12: RIPOR2 is expressed in the cytoplasm of melanocytes in benign melanocytic nevi.

Adjacent sections of a benign melanocytic nevus showing multinucleated cells and neuroid maturation, BRAF^{V600E} -positive. Dotted boxes are magnified in the adjacent panels. **A -** H&E stain shows tissue disorganization in the epidermis and dermis. **B** - Immunofluorescence staining with the anti-SOX10 shows SOX10 expression in melanocytes. Immunofluorescence staining with the anti-RIPOR2 antibodies demonstrates that, similar to the SOX10 melanocytes marker, RIPOR2 is expressed in the neuroid structure formed by transformed melanocytes in the dermis. White arrowheads point to RIPOR2+ multinucleated cells. Blue represents Hoechst nuclear staining in all panels. Scale bar: 100 µm.

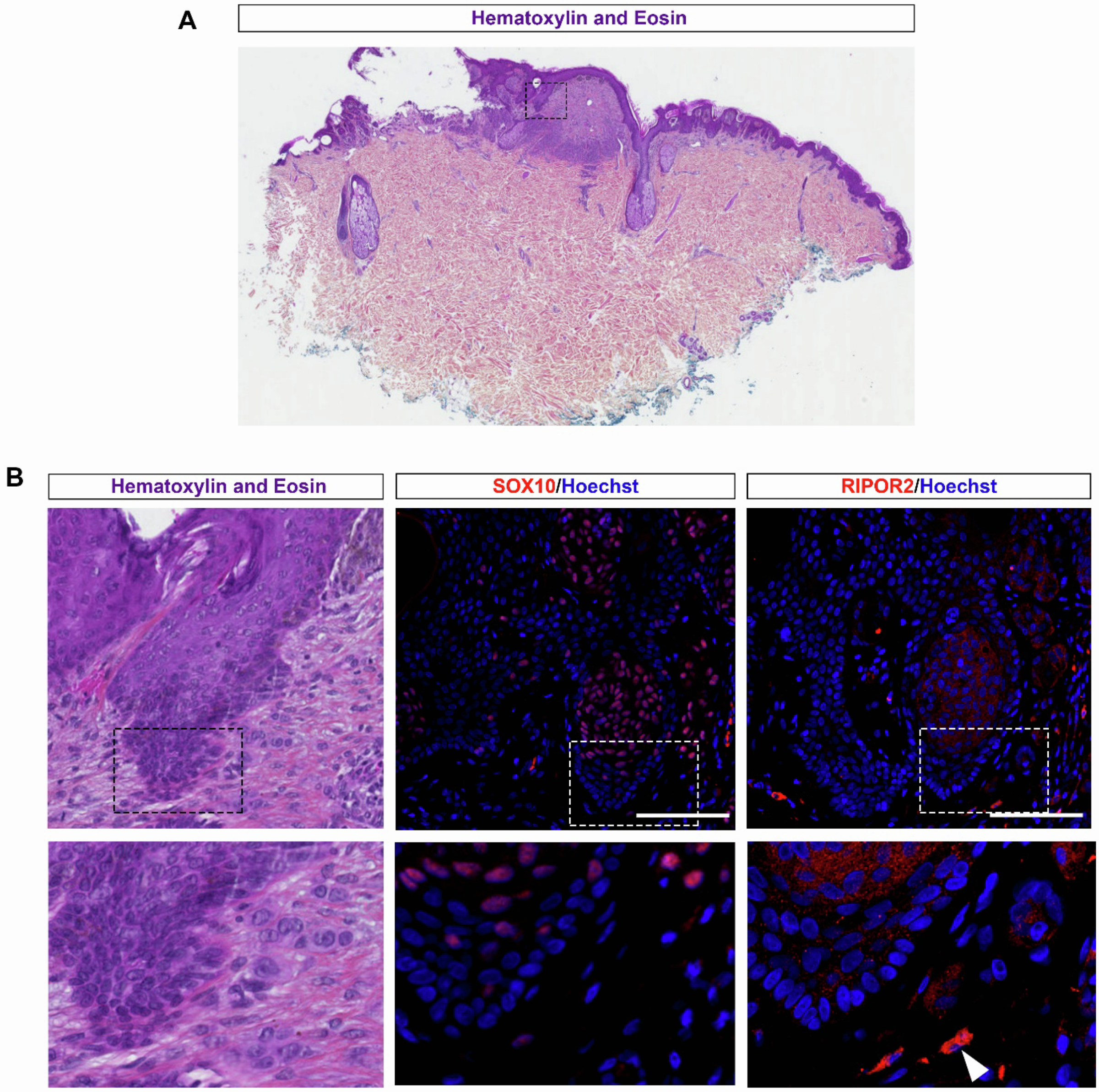

Supplementary Figure 13: RIPOR2 is expressed in the cytoplasm of melanocytes in intermediate melanocytic lesions.

Adjacent sections of a dysplastic melanocytic nevus, $BRAF^{V600E}$-positive. Dotted boxes are magnified in the adjacent panels. **A -** H&E stain shows tissue disorganization in the epidermis. **B -** Immunofluorescence with the anti-SOX10 and anti- RIPOR2 antibodies demonstrates that RIPOR2 is expressed in SOX10+ structures formed by transformed melanocytes in the epidermis. White arrowheads point to an immune cell expressing RIPOR2 (higher compared to melanocytes). Blue represents Hoechst nuclear staining in all panels. Scale bar: 100 µm.

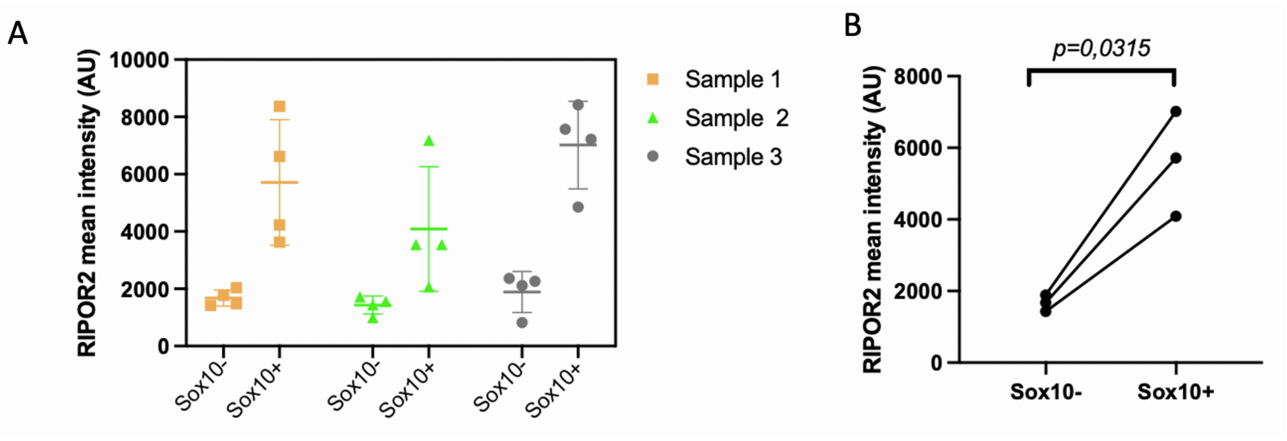

Supplementary Figure 14: Quantification of RIPOR2 immunofluorescence in human tissues.

A- For 3 samples of melanocytic lesions positive for BRAF^{V600E} staining of human skin, SOX10 positive (SOX10+) and SOX10 low (SOX10-) areas were defined on an adjacent section, and the mean intensity of RIPOR2 was measured on 4 ROI for each SOX10+/SOX10- area. B- A paired t-test was applied.

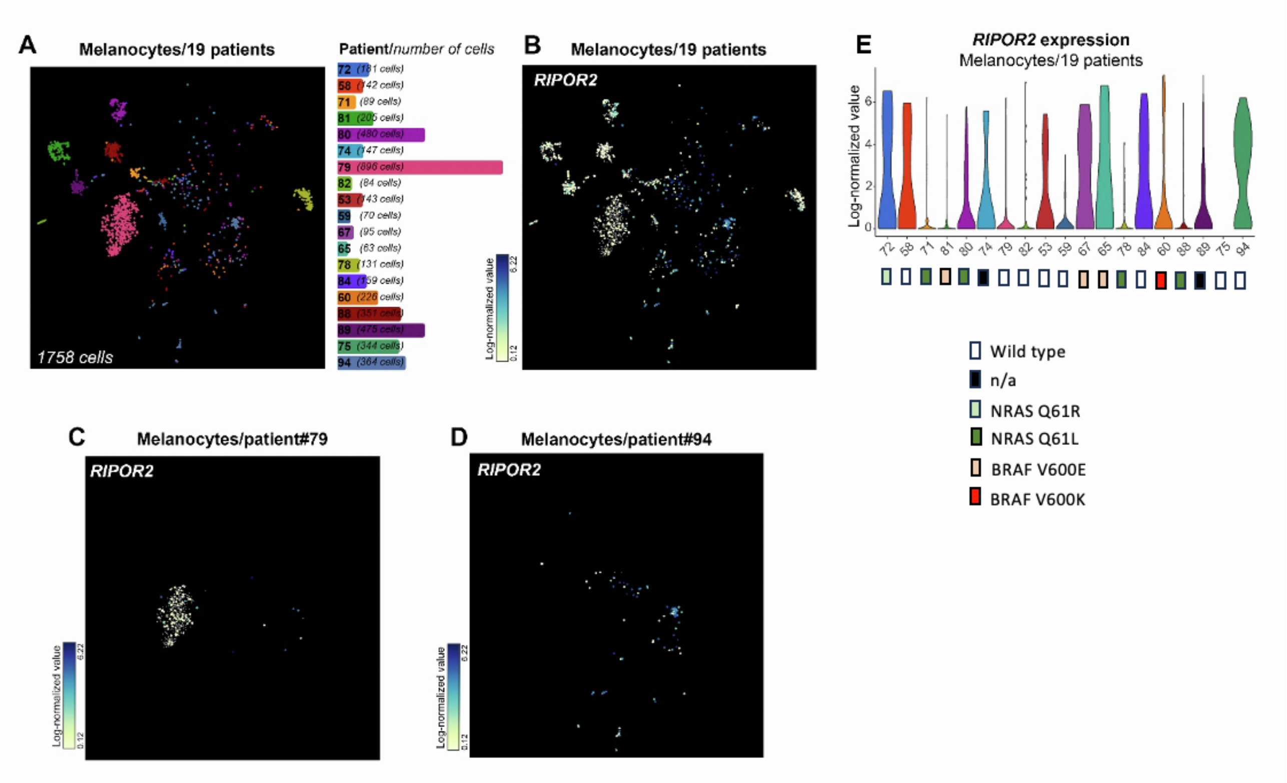

Supplementary Figure 15: *RIPOR2* is expressed in the melanocytes of most melanoma.

Single cell RNAseq analyses using BBrowser 2 and the transcriptomic data from 19 melanoma patients from Tirosh et al., 2016 (GSE72056). **A -** t-SNE (t-distributed stochastic neighbor embedding) visualization of melanocyte cells of the 19 patients and the corresponding colour code on the right. **B-C-D** Expression of *RIPOR2* at the single cell level in this data set shows that *RIPOR2* is expressed in some of the melanocytes of most patients, with some variations (patient#79 has only a few RIPOR2 + melanocytes compared to patient#94). **E -** Violin plot of RIPOR2 expression in the melanocytes of the 19 patients. The colored rectangles below the patient numbers indicate their mutation status. The violin plot illustrates that *RIPOR2* is expressed in melanocytes of nearly all the patients (18/19), with strong variation, regardless of their mutation status.

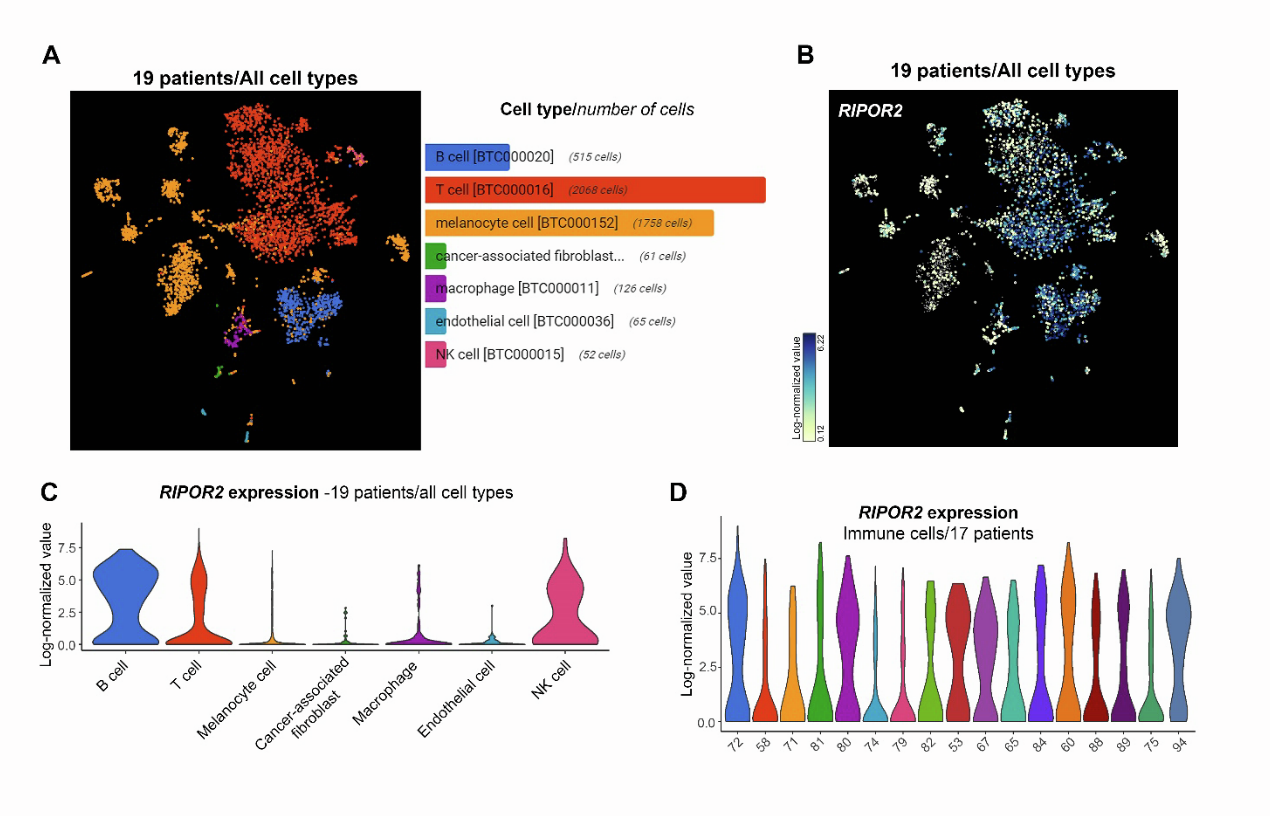

Supplementary Figure 16: *RIPOR2* is highly expressed in blood/immune cells.

Single cell RNAseq analyses using BBrowser 2 and the transcriptomic data from 19 melanoma patients from Tirosh et al., 2016 (GSE72056). **A -** t-SNE visualization of all the cell types of the 19 patients and the corresponding colour code on the right. **B** – Expression of *RIPOR2* at the single cell level in this data set. **C -** Violin plot of *RIPOR2* expression in the 19 patients for different cell types shows that *RIPOR2* is highly expressed in immune cells (T cells, B cells, macrophage, and NK cells). **D -**Violin plot of *RIPOR2* expression in the immune cells of the 17 patients show that *RIPOR2* is expressed with equivalent levels of expression among them.

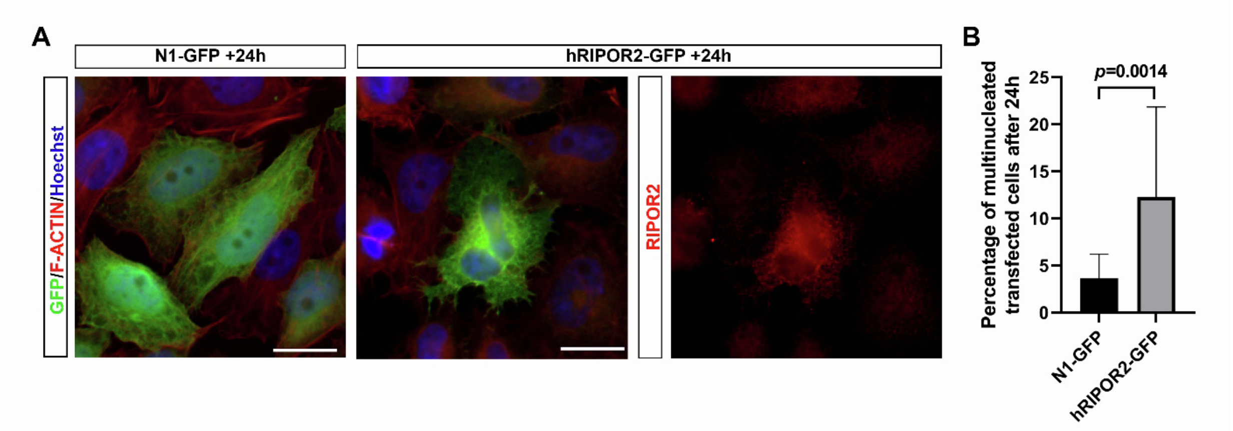

Supplementary Figure 17: Transient transfection of Hela cells with a vector expressing human RIPOR2 induces an increase in the number of multinucleated cells.

A – Immunofluorescence staining with the anti-GFP and anti-RIPOR2 antibodies and F-ACTIN staining in HeLa cell line, transfected with either a control plasmid expressing only GFP (N1-GFP) or human RIPOR2-GFP (hRIPOR2-GFP) for 24 hours. The transitory expression of h RIPOR2 increases the number of transfected (GFP+) multinucleated cells, quantify in **B -** represented as the percentage of transfected multinucleated cells (N1-GFP: 559 cells; hRIPOR2: 595 cells; 5 independent experiments, Fisher's exact test). Blue represents Hoechst nuclear staining. Scale bar: 20 µm.

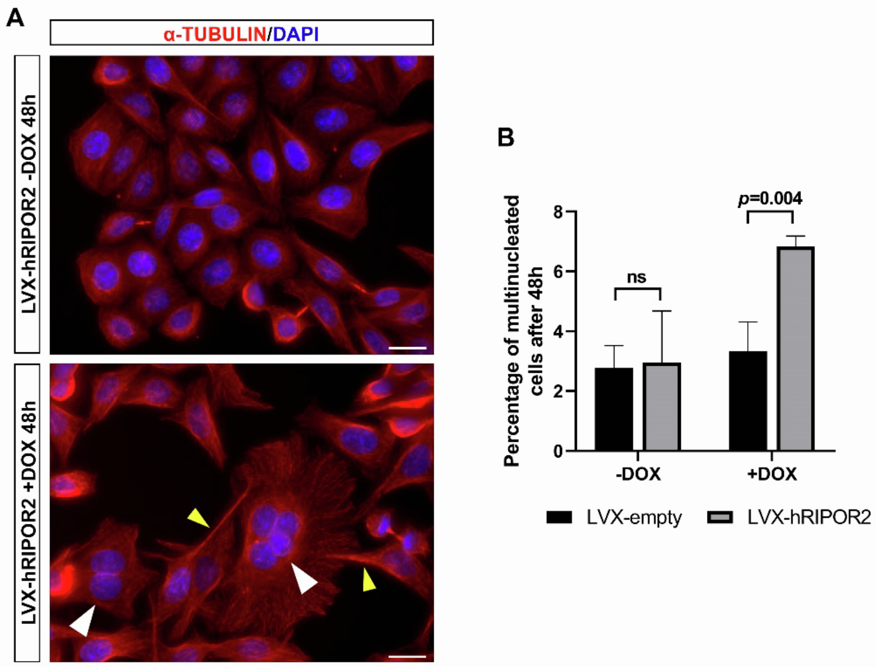

Supplementary Figure 18: Doxycycline induction of RIPOR2 expression in Hela cell line induces an increase in the number of multinucleated cells.

A - α-TUBULIN staining in the HeLa cell line, stably infected with doxycycline-inducible plasmid (LVX-TetOne-Puro) expressing either only GFP (LVX-GFP) or human RIPOR2 (LVX-hRIPOR2). The two cell lines were incubated with doxycycline (+DOX) or in the absence of doxycycline (-DOX, control) for 48 hours. The stable expression of hRIPOR2 induces cellular protrusions (yellow arrowheads) and increases the number of multinucleated cells (white arrowheads), quantified in **B -** represented as the percentage of multinucleated cells (LVX-GFP -DOX: 711 cells, LVX-GFP +DOX: 659 cells, LVX-hRIPOR2 -DOX: 635 cells, LVX- hRIPOR2 +DOX: 643 cells; 3 independent experiments, Fisher's exact test). Blue represents Hoechst nuclear staining. Scale bar: 20 µm.

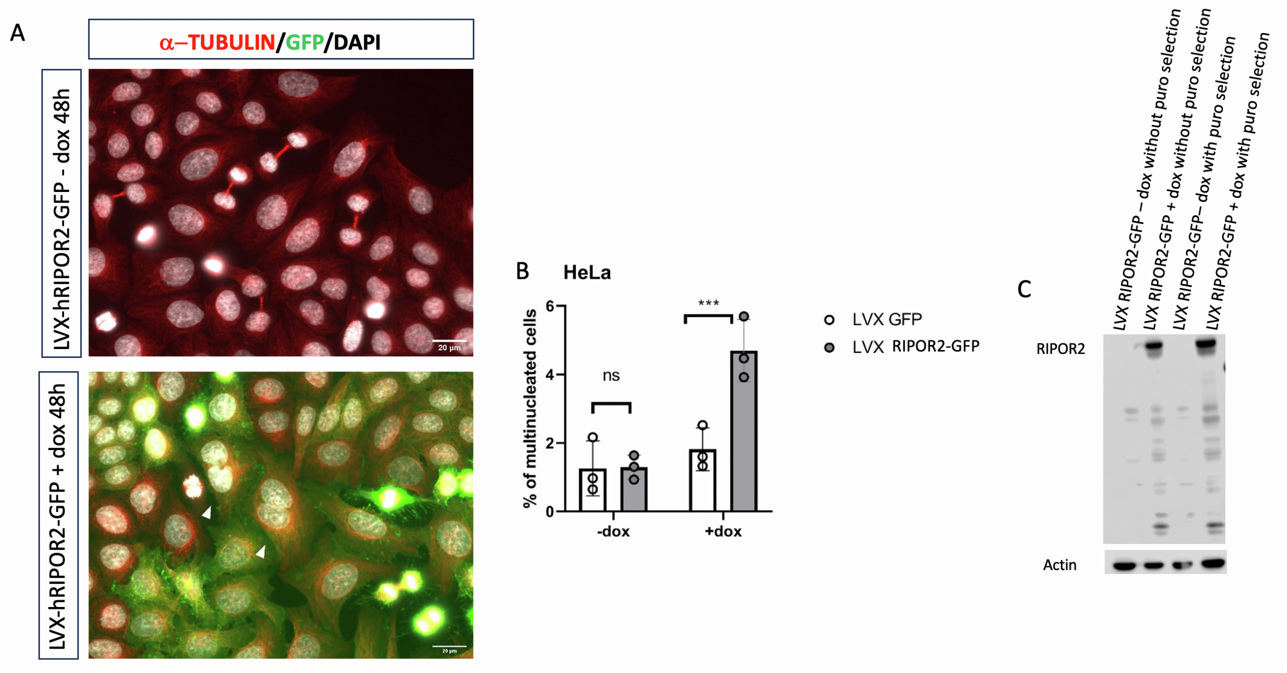

Supplementary Figure 19: Doxycycline induction of RIPOR2-GFP expression in Hela cell line induces an increase in the number of multinucleated cells.

A - α-TUBULIN and GFP staining in HeLa cells, stably infected with doxycycline-inducible plasmid (LVX-TetOne-Puro) expressing human RIPOR2-GFP fusion protein (LVX-hRIPOR2-GFP). The cells were incubated with doxycycline (+DOX) or without doxycycline (-DOX, control) for 48 hours. The stable expression of hRIPOR2-GFP induces an increase in the number of multinucleated cells (white arrowheads), quantified in **B -** represented as the percentage of multinucleated cells (>80 GFP positive cells for each condition; 3 independent experiments, Fisher's exact test, *** < 0.001). **C –** Western blot for RIPOR2 and for Actin confirm the induction of RIPOR2-GFP after 24 hours with Doxycycline. Scale bar: 20 µm.
