# Supplementary material for: RIPOR2 promotes multinucleation of melanoma cells downstream of the RAS/ERK oncogenic pathway
Source: iScience. 2026 Apr 15;29(5):115734. doi: 10.1016/j.isci.2026.115734 (PMC13145898; doi:10.1016/j.isci.2026.115734)
Supplement: Table S1. List of MEK1ca versus control DGEs among GFP-positive cells [file mmc2.pdf]

Table S1: List of MEK1ca versus control DGEs among GFP-positive cells

List of MEK1ca versus control DGEs among GFP-positive cells – Upregulated genes:

FeatureID	FeatureName	GFP+sup1 MEK1ca Average	GFP+sup1 MEK1ca Log2 Fold Change	GFP+sup1 MEK1ca P-Value	GFP+sup1 Control Average	GFP+sup1 Control Log2 Fold Change	GFP+sup1 Control P-Value
**feature_5174**	ENSGALG00000007656	1.7321005688984064	5.241964768631123	1.765833266532058e-14	0.03824831148431099	-5.241964768631123	1.765833266532058e-14
**feature_8902**	CHST15	2.8290975958673967	4.13871961258768	1.765833266532058e-14	0.15299324593724395	-4.13871961258768	1.765833266532058e-14
**feature_1621**	IL1R1	1.47805915212664	4.429824784008142	1.4509693273596444e-11	0.06119729837489758	-4.429824784008142	1.4509693273596444e-11
**feature_6794**	PRR16	2.251730739567928	3.8813042792550463	1.4509693273596444e-11	0.14534358364038175	-3.8813042792550463	1.4509693273596444e-11
**feature_11903**	PTPRT	2.9792129785052586	4.435405816271442	1.9301077986497985e-11	0.13004425904665737	-4.435405816271442	1.9301077986497985e-11
**feature_3141**	ENSGALG00000013624	1.143186375472948	5.232378719801925	4.622781089706883e-10	0.02294898689058659	-5.232378719801925	4.622781089706883e-10
**feature_10495**	IL17RD	2.436488133583758	3.031040765728151	5.864674035529186e-09	0.2906871672807635	-3.031040765728151	5.864674035529186e-09
**feature_2667**	UBE2E2	12.251724690674727	2.2064623531946292	2.8900030533432485e-08	2.639133492417458	-2.2064623531946292	2.8900030533432485e-08
**feature_368**	KCNC2	2.344109436575843	2.9755456531364475	3.466158455570338e-08	0.2906871672807635	-2.9755456531364475	3.466158455570338e-08
**feature_7897**	TFPI	4.087757342600239	3.6678803216404843	4.411066923667722e-08	0.3136361541713501	-3.6678803216404843	4.411066923667722e-08
**feature_4249**	PRKN	9.2378697007915	3.519935444769788	1.0322258324906826e-07	0.7955648788736686	-3.519935444769788	1.0322258324906826e-07
**feature_2336**	PTH2R	1.8129319287803318	3.0849483561466995	3.9905309981009377e-07	0.20654088201527934	-3.0849483561466995	3.9905309981009377e-07
**feature_138**	MAGI2	10.612002818784235	1.9993843533348512	9.092492347913337e-07	2.639133492417458	-1.9993843533348512	9.092492347913337e-07
**feature_10916**	ADAMTS18	1.9053106257882468	3.208674459210657	9.092492347913337e-07	0.19889121971841714	-3.208674459210657	9.092492347913337e-07
**feature_13734**	HOXB9	1.6859112203944489	2.1734850307483566	1.5677264452232596e-06	0.36718379024938547	-2.1734850307483566	1.5677264452232596e-06
**feature_869**	PACSIN2	2.575056179095631	2.3735096390563504	1.7314113845566705e-06	0.48957838699918066	-2.3735096390563504	1.7314113845566705e-06
**feature_5627**	SPATA5	5.184754369569229	1.8945090810455416	4.076020966452151e-06	1.3845888757320577	-1.8945090810455416	4.076020966452151e-06
**feature_4698**	PXDN	2.9214762928753117	2.0536452607423534	8.514448256586862e-06	0.69611926901446	-2.0536452607423534	8.514448256586862e-06
**feature_2630**	HOXA7	1.351038443740757	2.3418825624440753	1.1148453842778002e-05	0.26008851809331474	-2.3418825624440753	1.1148453842778002e-05
**feature_776**	WNT5B	2.7829082473634394	2.7260260537771352	3.1911849475953196e-05	0.4130817640305587	-2.7260260537771352	3.1911849475953196e-05
**feature_7615**	NRXN3	3.972283971340345	3.3465497397212363	4.424524939330474e-05	0.3824831148431099	-3.3465497397212363	4.424524939330474e-05
**feature_2371**	SCN5A	1.7667425802763743	4.047954148664498	4.87285325563697e-05	0.09944560985920857	-4.047954148664498	4.87285325563697e-05
**feature_7737**	KIF26A	1.5819851862605443	2.5271219853630575	7.570797096497341e-05	0.26773818039017694	-2.5271219853630575	7.570797096497341e-05
**feature_2917**	JARID2	7.75981054866486	1.4751905844388213	0.00015088452103014335	2.776827413760978	-1.4751905844388213	0.00015088452103014335
**feature_1985**	ENSGALG00000019077	1.7090058946464275	2.1352657085178666	0.0001695639173914887	0.3824831148431099	-2.1352657085178666	0.0001695639173914887
**feature_3429**	RALYL	1.9053106257882468	3.00936565098725	0.00025336186584748314	0.22948986890586592	-3.00936565098725	0.00025336186584748314
**feature_1724**	EFNB2	1.258659746732842	2.0123302389337767	0.0006465746329067641	0.3059864918744879	-2.0123302389337767	0.0006465746329067641
**feature_4402**	PTPRK	8.30253539358636	1.4185975285848884	0.0006495088012736354	3.090463567932328	-1.4185975285848884	0.0006495088012736354
**feature_8285**	MYLK	1.6050798605125232	1.7870682094092807	0.0009011228847809678	0.45897973781173185	-1.7870682094092807	0.0009011228847809678
**feature_10222**	IGF1R	5.103923009687303	1.2555641067530312	0.0009012045818070775	2.126606118527691	-1.2555641067530312	0.0009012045818070775
**feature_9698**	MB21D2	1.258659746732842	1.910450624914563	0.0009070393605223946	0.3289354787650745	-1.910450624914563	0.0009070393605223946
**feature_416**	ATP2B1	2.274825413819907	1.5036336324406876	0.0009260887663047164	0.7955648788736686	-1.5036336324406876	0.0009260887663047164
**feature_9676**	DLG1	4.734408221655643	1.294237190208967	0.001384634607494714	1.9200652365124116	-1.294237190208967	0.001384634607494714
**feature_12241**	TMEM132C	8.175514685200477	1.4434545031515083	0.001449685421376066	2.9910179580731193	-1.4434545031515083	0.001449685421376066
**feature_5110**	PCDH19	1.0277130042130544	1.951092609411909	0.0016928100014335945	0.26008851809331474	-1.951092609411909	0.0016928100014335945
**feature_5322**	TLL1	2.2979200880718857	2.103095702856959	0.0017148804671323042	0.5278266984834916	-2.103095702856959	0.0017148804671323042
**feature_3839**	SPRED2	3.5912218461826955	1.3661301086907525	0.001857408249441927	1.3845888757320577	-1.3661301086907525	0.001857408249441927
**feature_3042**	MARCHF6	1.5473431748825763	1.4174106136344458	0.0022907661018388083	0.5737246722646648	-1.4174106136344458	0.0022907661018388083
**feature_10266**	SMAD3	2.5519615048436517	1.4404238910380665	0.003164399346788044	0.9332588002171881	-1.4404238910380665	0.003164399346788044
**feature_6718**	VCAN	5.935331282758539	1.4780100793742736	0.0032443991279739836	2.118956456230829	-1.4780100793742736	0.0032443991279739836
**feature_10824**	SALL1	1.3972277922447145	1.5192598675900868	0.0034284887657424954	0.48192872470231846	-1.5192598675900868	0.0034284887657424954
**feature_2695**	TRIM71	3.8106212515764937	1.3225853162907693	0.003520966886941413	1.5146331347787152	-1.3225853162907693	0.003520966886941413
**feature_1493**	SCML2	1.1893757239769056	1.5885225300272006	0.004102573110260808	0.3901327771399721	-1.5885225300272006	0.004102573110260808
**feature_1792**	NDFIP2	1.7090058946464275	1.485762955602	0.004346943863935078	0.6043233214521136	-1.485762955602	0.004346943863935202
**feature_4422**	FABP7	1.7321005688984064	1.8269272693522793	0.005005105322955952	0.48192872470231846	-1.8269272693522793	0.005005105322955952
**feature_14227**	HOXC10	1.1316390383469588	1.82556172732805	0.005005105322955952	0.3136361541713501	-1.82556172732805	0.005005105322955952
**feature_4120**	CRIM1	3.6720532060646214	1.6298107385178322	0.005360523341583206	1.1780479937167785	-1.6298107385178322	0.005360523341583206
**feature_1663**	RASA3	1.8360266030323107	1.3405950165836151	0.007440470347852847	0.7190682559050466	-1.3405950165836151	0.007440470347852847
**feature_6247**	RAI14	1.7436479060243957	1.646625484590769	0.0080250248568461	0.5507756853740783	-1.646625484590769	0.0080250248568461
**feature_4735**	GREB1	3.452653800670823	1.1662895293441526	0.008413644635060726	1.5299324593724395	-1.1662895293441526	0.008413644635060726
**feature_3083**	FHOD3	9.780594545713	1.0828233078306972	0.01085967267869174	4.597447040414181	-1.0828233078306972	0.01085967267869174
**feature_2842**	VOPP1	1.2355650724808631	1.485429037063097	0.012339803599224996	0.4360307509211453	-1.485429037063097	0.012339803599224996
**feature_3445**	MMP16	3.3371804294109295	1.3090000014048375	0.013973594106901405	1.3386909019508846	-1.3090000014048375	0.013973594106901405
**feature_3873**	PLCB1	3.475748474922802	1.234470232084199	0.014110585570077027	1.4687351609975419	-1.234470232084199	0.014110585570077027
**feature_11343**	SPOCK1	1.5357958377565868	1.654611720484973	0.02031527758980733	0.48192872470231846	-1.654611720484973	0.02031527758980733
**feature_2965**	ENSGALG00000012808	2.702076887481514	1.1431113817048377	0.020524280322963814	1.2162963052010893	-1.1431113817048377	0.020524280322963814
**feature_11281**	GRIA1	1.0623550155910224	2.604119385078219	0.02185190715777829	0.16829257053096836	-2.604119385078219	0.02185190715777829
**feature_2626**	HOXA3	8.418008764846254	0.9378838550376756	0.025037657219290438	4.375606833805177	-0.9378838550376756	0.025037657219290438
**feature_1642**	LIMS1	2.7482662359854713	1.176506382723594	0.029919911239280696	1.2086466429042273	-1.176506382723594	0.029919911239280696
**feature_11155**	TENM2	10.70438151579215	1.1133481748441563	0.03241409735078604	4.926382519179255	-1.1133481748441563	0.03241409735078604
**feature_2684**	RBMS3	14.249414013470888	0.8844032666937132	0.03494079871595575	7.687910608346509	-0.8844032666937132	0.03494079871595575
**feature_4470**	REV3L	3.972283971340345	0.9855520801552817	0.03971586878480132	1.9965618594810337	-0.9855520801552817	0.03971586878480132
**feature_6486**	MLLT3	2.1131626940560557	1.089716673055759	0.04412849665121076	0.9868064362952235	-1.089716673055759	0.04412849665121076
**feature_7900**	ITGAV	1.0623550155910224	1.542718840414076	0.04779191501832291	0.3595341279525233	-1.542718840414076	0.04779191501832291
**feature_7701**	BEGAIN	1.2124703982288845	3.8570113659531025	0.05318487101086248	0.07649662296862197	-3.8570113659531025	0.05318487101086248
**feature_654**	MYH9	1.2124703982288845	1.10698961896145	0.0748012302798907	0.5584253476709404	-1.10698961896145	0.0748012302798907
**feature_4316**	ARID1B	3.752884565946547	0.9714663995961912	0.0795336680461184	1.9047659119186873	-0.9714663995961912	0.0795336680461184
**feature_8800**	SLK	1.8129319287803318	1.1918635600632113	0.0899702149481121	0.7879152165768064	-1.1918635600632113	0.0899702149481121
**feature_5839**	PCDH7	3.568127171930717	0.9942822157180337	0.09068945300251866	1.782371315168892	-0.9942822157180337	0.09068945300251866
**feature_809**	PTPRO	1.2471124096068524	2.2692440135537875	0.092181186667444	0.25243885579645253	-2.2692440135537875	0.092181186667444
**feature_3624**	KCNQ3	2.436488133583758	1.496264022175212	0.092181186667444	0.8567621772485662	-1.496264022175212	0.092181186667444
**feature_722**	KDM7A	1.1547337125989374	1.056909453898978	0.09230702729062344	0.5507756853740783	-1.056909453898978	0.09230702729062344
**feature_7898**	CALCRL	2.852192270119376	1.333265474785126	0.09313378391257962	1.1245003576387431	-1.333265474785126	0.09313378391257962
**feature_6801**	SEMA6A	4.642029524647729	1.1987644391967145	0.10291504337787934	2.011861184074758	-1.1987644391967145	0.10291504337787934
**feature_12155**	TSHZ2	4.3071567479940365	1.0693277994570647	0.10956337298819159	2.042459833262207	-1.0693277994570647	0.10956337298819159
**feature_8811**	ADD3	1.2817544209848206	1.0035600293060445	0.12377941000098264	0.6349219706395625	-1.0035600293060445	0.12377941000098264
**feature_7442**	SIPA1L1	1.385680455118725	1.3374607658594826	0.12558073088774088	0.543126023077216	-1.3374607658594826	0.12558073088774088
**feature_125**	SEMA3A	2.21708872818996	1.2267832569084056	0.13422891962430974	0.9409084625140504	-1.2267832569084056	0.13422891962430974
**feature_2813**	PDE1C	1.6166271976385125	1.2523404514595962	0.1444828167482304	0.6731702821238734	-1.2523404514595962	0.1444828167482304
**feature_1529**	ARHGAP6	1.2702070838588313	1.5002953470154652	0.16422890187595515	0.44368041321800744	-1.5002953470154652	0.16422890187595515
**feature_16040**	UHRF1	1.3972277922447145	0.9045500234748785	0.1801865866618177	0.7420172427956332	-0.9045500234748785	0.18018658666182025
**feature_9367**	LRP8	2.2632780766939176	0.8976913941992215	0.18033230592478475	1.2086466429042273	-0.8976913941992215	0.18033230592478475
**feature_3264**	ZNF521	11.0854436409498	0.7337027839865509	0.20544078830786186	6.639906873676388	-0.7337027839865509	0.20544078830786186
**feature_8155**	LRP2	1.4434171407486718	0.9658896113399893	0.2293027134455628	0.7343675804987709	-0.9658896113399893	0.2293027134455628
**feature_3140**	CARMIL1	1.8360266030323107	0.7607035054098809	0.23117614662912522	1.0786023838575698	-0.7607035054098809	0.23117614662912497
**feature_3217**	PTPRM	3.7644319030725364	0.9416693555252832	0.24483351860518351	1.9506638856998604	-0.9416693555252832	0.24483351860518351
**feature_7181**	OSBPL5	1.166281049724927	1.2836679484987799	0.2544801036795234	0.47427906240545625	-1.2836679484987799	0.2544801036795234
**feature_3246**	GREB1L	2.8406449329933863	0.775338529167399	0.26184032197685736	1.6523270561222347	-0.775338529167399	0.26184032197685736
**feature_6623**	PAM	1.3394911066147674	0.9353252935535922	0.2645741567112692	0.69611926901446	-0.9353252935535922	0.2645741567112692
**feature_4158**	CHRM3	1.351038443740757	1.167384831211939	0.26635778492170825	0.5966736591552514	-1.167384831211939	0.26635778492170825
**feature_7156**	PIK3C2A	1.258659746732842	0.8307234324438288	0.27065840419359993	0.7037689313113222	-0.8307234324438288	0.27065840419359993
**feature_6433**	SMARCA2	1.4896064892526293	0.9670341532809302	0.27558788236399956	0.7573165673893576	-0.9670341532809302	0.27558788236399956
**feature_6286**	GHR	1.073902352717012	0.993364262200156	0.27728071221630335	0.5354763607803539	-0.993364262200156	0.27728071221630335
**feature_3973**	EPRS	1.10854436409498	0.8308833675962446	0.2849244810084043	0.619622646045838	-0.8308833675962446	0.2849244810084043
**feature_4138**	KIF26B	1.0392603413390438	1.1421208598390216	0.2849244810084043	0.46662940010859405	-1.1421208598390216	0.2849244810084043
**feature_3012**	CDH6	4.699766210277676	1.396761727344416	0.2873775290399689	1.7747216528720298	-1.396761727344416	0.2873775290399689
**feature_725**	MKRN4P	2.147804705434024	0.7690947756690215	0.29207062301944015	1.2545446166854004	-0.7690947756690215	0.29207062301944015
**feature_13047**	FNBP1	1.3741331179927356	0.8372016428839244	0.2935616631451051	0.7649662296862197	-0.8372016428839244	0.2935616631451051
**feature_9088**	RALGPS2	1.050807678465033	0.8266822672219649	0.29899335756524503	0.5890239968583892	-0.8266822672219649	0.29899335756524503
**feature_5563**	ADGRL3	9.018470295397702	0.654582894545316	0.3051412890860803	5.706648073459199	-0.654582894545316	0.3051412890860803
**feature_14547**	ENSGALG00000031355	1.7321005688984064	0.8269272693522794	0.3221908498548448	0.9715071117014991	-0.8269272693522794	0.3221908498548448
**feature_8358**	KIF5C	1.10854436409498	0.8485853693297035	0.3261881753391393	0.6119729837489758	-0.8485853693297035	0.3261881753391393
**feature_308**	LRIG3	1.4549644778746613	0.9624973726841581	0.32886190160480777	0.7420172427956332	-0.9624973726841581	0.32886190160480777
**feature_16394**	ENSGALG00000050132	82.71357583346189	0.5525253765695197	0.37055257880218967	56.17911990815598	-0.5525253765695197	0.37055257880204723
**feature_5172**	ARHGEF9	1.2817544209848206	0.986486515947103	0.3771739316813683	0.6425716329364246	-0.986486515947103	0.3771739316813683
**feature_14230**	HOXC6	5.080828335435325	0.6350651159642311	0.3860149509533673	3.2587561384632964	-0.6350651159642311	0.3860149509533673
**feature_9614**	EPHA4	1.4318698036226825	0.867806287506069	0.4088245616637982	0.7802655542799442	-0.867806287506069	0.4088245616637982
**feature_5704**	ENSGALG00000046550	2.9099289557493226	0.913304621969716	0.41333052896162614	1.5375821216693017	-0.913304621969716	0.41333052896162614
**feature_1747**	CLYBL	1.5011538263786188	0.9354450043814324	0.41730928449984633	0.7802655542799442	-0.9354450043814324	0.41730928449984633
**feature_10049**	TLN2	2.055426008426109	0.6629473711537553	0.4296855589994978	1.2927929281697115	-0.6629473711537553	0.4296855589994978
**feature_8349**	LRP1B	4.122399353978207	2.0723383072914574	0.4330141530815264	0.9715071117014991	-2.0723383072914574	0.4330141530815264
**feature_10043**	USP3	1.4318698036226825	0.6963258195617154	0.4330141530815264	0.8797111641391527	-0.6963258195617154	0.4330141530815264
**feature_6382**	LHFPL2	2.40184612220579	0.7109191613869272	0.46808345406074403	1.4610854987006798	-0.7109191613869272	0.46808345406074403
**feature_3529**	RIMS2	1.0854496898430013	0.8545973901810456	0.4859570655407906	0.5966736591552514	-0.8545973901810456	0.4859570655407906
**feature_10932**	CMIP	1.143186375472948	0.7566452888355275	0.4859570655407906	0.6731702821238734	-0.7566452888355275	0.4859570655407906
**feature_2411**	ADARB2	1.5588905120085657	1.1681907310788437	0.4938492750365357	0.6884696067175978	-1.1681907310788437	0.4938492750365357
**feature_7894**	COL5A2	1.6281745347645018	0.7495849627597168	0.5060555818069258	0.9638574494046369	-0.7495849627597168	0.5060555818069258
**feature_2063**	MAML2	4.9422602899234525	0.6823046763478178	0.5399237655543487	3.0675145810417415	-0.6823046763478178	0.5399237655543487

**feature_4244**	PDE10A	3.9607366342143555	0.6224698619505382	0.5399237655543487	2.5626368694488364	-0.6224698619505382	0.5399237655543487
**feature_397**	TMTC2	3.360275103662908	0.6336888460753354	0.5402962028047148	2.1572047677151396	-0.6336888460753354	0.5402962028047148
**feature_10480**	BICD2	1.073902352717012	0.6673779507384402	0.5426054641719854	0.6731702821238734	-0.6673779507384402	0.5426054641719874
**feature_7148**	PDE3B	1.0854496898430013	0.7489872022204469	0.5553590888131095	0.6425716329364246	-0.7489872022204469	0.5553590888131095
**feature_9414**	DOCK7	1.4087751293707038	0.6483939860045993	0.5553590888131095	0.8950104887328771	-0.6483939860045993	0.5553590888131095
**feature_361**	LGR5	1.1778283868509163	0.8827056344316587	0.5561948974811434	0.6349219706395625	-0.8827056344316587	0.5561948974811434
**feature_7324**	HSD17B12	1.2471124096068524	0.6702063276209086	0.5623205880629163	0.7802655542799442	-0.6702063276209086	0.5623205880629163
**feature_6514**	PCSK5	2.0438786713001194	0.9874403639427676	0.5804156834794134	1.0250547477795344	-0.9874403639427676	0.5804156834794134
**feature_115**	FAM107B	1.166281049724927	1.032129181502815	0.5994813068732763	0.5660750099678026	-1.032129181502815	0.5994813068732763
**feature_2631**	HOXA9	1.6512692090164807	0.6188961790707195	0.6423407526915823	1.0709527215607078	-0.6188961790707195	0.6423407526915823
**feature_10543**	SLC6A6	1.073902352717012	0.8032613788202133	0.6444157613839704	0.6119729837489758	-0.8032613788202133	0.6444157613839704
**feature_2799**	sept-07	1.4203224664966931	0.5885225300272006	0.6560308759974934	0.9409084625140504	-0.5885225300272006	0.6560308759974934
**feature_6364**	MAST4	2.528866830591673	0.5754663772017543	0.6602047266902573	1.6905753676065456	-0.5754663772017543	0.6602047266902573
**feature_8356**	MBD5	1.385680455118725	0.6247427179399538	0.6990768117625289	0.8950104887328771	-0.6247427179399538	0.6990768117625289
**feature_740**	PTN	1.7667425802763743	0.6954377339437127	0.7040632246762859	1.086252046154432	-0.6954377339437127	0.7040632246762885
**feature_4015**	UBR2	1.0854496898430013	0.5885225300272008	0.7078904248191736	0.7190682559050466	-0.5885225300272008	0.7078904248191736
**feature_10488**	CACNA2D3	1.200923061102895	1.017365828831075	0.7132069713714664	0.5890239968583892	-1.017365828831075	0.7132069713714664
**feature_5519**	ANTXR2	1.3394911066147674	0.5400240123360105	0.7170374520374384	0.9179594756234637	-0.5400240123360105	0.7170374520374384
**feature_9976**	NPTN	1.29330175811081	0.5758114782776466	0.7367548862917601	0.8644118395454283	-0.5758114782776466	0.7367548862917601
**feature_3544**	HSF1	1.3163964323627888	0.6526528674469162	0.7437505284788986	0.8338131903579795	-0.6526528674469162	0.7437505284788986
**feature_11371**	ENSGALG00000029896	1.385680455118725	0.5885225300272005	0.751999165904208	0.9179594756234637	-0.5885225300272005	0.751999165904208
**feature_2666**	ENSGALG00000011283	2.8290975958673967	0.636219272058497	0.7646585053301032	1.8129699643563408	-0.636219272058497	0.7646585053301032
**feature_663**	LARGE1	1.8706686144102789	0.6334699360811751	0.7777896831844019	1.200996980607365	-0.6334699360811751	0.7777896831844019
**feature_13145**	DAB2IP	1.073902352717012	0.6836797630675406	0.7777896831844019	0.6655206198270112	-0.6836797630675406	0.7777896831844019
**feature_1309**	APP	2.8290975958673967	0.5198097799431863	0.7858766544186221	1.965963210293585	-0.5198097799431863	0.7858766544186221
**feature_8827**	TCF7L2	4.4919141420098665	0.4409653416133428	0.81620695457732	3.297004449947607	-0.4409653416133428	0.81620695457732
**feature_784**	CACNA1C	1.9976893227961618	0.6651438116301133	0.8186013434373186	1.2545446166854004	-0.6651438116301133	0.8186013434373186
**feature_6335**	PDE4D	1.755195243150385	0.6860390359414631	0.8271506187226548	1.086252046154432	-0.6860390359414631	0.8271506187226548
**feature_3652**	ADGRB1	1.6050798605125232	0.6517163565143945	0.8332676237203918	1.0174050854826724	-0.6517163565143945	0.8332676237203918
**feature_2979**	EXOC2	1.570437849134555	0.49673005410771015	0.834048499396882	1.1092010330450186	-0.49673005410771015	0.834048499396882
**feature_6524**	TLE4	1.4318698036226825	0.6001105043024122	0.840867363209488	0.9409084625140504	-0.6001105043024122	0.840867363209488
**feature_7896**	GULP1	1.050807678465033	0.5422288777532656	0.8409036483863631	0.7190682559050466	-0.5422288777532656	0.8409036483863631
**feature_2627**	HOXA4	1.0277130042130544	0.5257867746792381	0.8424718717739209	0.7114185936081844	-0.5257867746792381	0.8424718717739209
**feature_5185**	NEXMIF	1.570437849134555	0.49673005410771015	0.8664766883834575	1.1092010330450186	-0.49673005410771015	0.8664766883834575
**feature_8119**	OLA1	1.5473431748825763	0.5257867746792378	0.8666380307564511	1.0709527215607078	-0.5257867746792378	0.8666380307564511
**feature_336**	CAND1	1.5473431748825763	0.4558847614490817	0.8670314602036451	1.1245003576387431	-0.4558847614490817	0.8670314602036451
**feature_4191**	SIPA1L2	1.7782899174023639	0.5247969324168134	0.8771379456796496	1.2315956297948139	-0.5247969324168134	0.8771379456796496
**feature_8268**	GLI2	5.473437797718963	0.4359121138870572	0.8833664616363398	4.031372030446378	-0.4359121138870572	0.8833664616363398
**feature_1753**	DOCK9	1.143186375472948	1.0035600293060443	0.9053388350746838	0.5660750099678026	-1.0035600293060443	0.9053388350746838
**feature_4020**	EML4	3.972283971340345	0.47208062180509525	0.9053388350746838	2.8533240367295996	-0.47208062180509525	0.9053388350746838
**feature_9513**	ENSGALG00000006724	2.182446716811992	0.7071670265258195	0.9053388350746838	1.3310412396540223	-0.7071670265258195	0.9053388350746838
**feature_331**	HMGA2	4.80369224441158	0.4220613838884595	0.9086348466461414	3.5723922926346465	-0.4220613838884595	0.9086348466461414
**feature_540**	TCF20	1.4665118150006506	0.4489711776284071	0.9179395177176818	1.0709527215607078	-0.4489711776284071	0.9179395177176818
**feature_4105**	TTC27	1.0969970269689906	0.47304531260726446	0.9197060878126593	0.7879152165768064	-0.47304531260726446	0.9197060878126593
**feature_3238**	YES1	1.3048490952367995	0.46721623380506705	0.9232922154804409	0.9409084625140504	-0.46721623380506705	0.9232922154804409
**feature_16660**	COX3	1.0277130042130544	0.5105200180259276	0.9232922154804409	0.7190682559050466	-0.5105200180259276	0.9232922154804409
**feature_1589**	TMEM131	1.4665118150006506	0.4489711776284071	0.9317525358158337	1.0709527215607078	-0.4489711776284071	0.9317525358158469
**feature_2639**	CREB5	1.2471124096068524	0.6152398684029801	0.9428705211888994	0.810864203467393	-0.6152398684029801	0.9428705211888994
**feature_7722**	EIF5	1.9976893227961618	0.4241357121263184	0.949700388200282	1.4840344855912664	-0.4241357121263184	0.9497003882002786
**feature_10504**	FLNB	1.0046183299610756	0.8000266352209122	0.9547606719426924	0.5737246722646648	-0.8000266352209122	0.9547606719426924
**feature_5599**	MOB1B	1.0623550155910224	0.4552559991637363	0.9575772973885456	0.772615891983082	-0.4552559991637363	0.9575772973885456
**feature_11359**	DDX46	2.58660351622162	0.39107646582098343	0.9579382633448882	1.965963210293585	-0.39107646582098343	0.9579382633448814
**feature_13180**	MAPKAP1	1.3394911066147674	0.42546424807315486	0.9637941845783162	0.9944560985920857	-0.42546424807315486	0.9637941845783162
**feature_2623**	SKAP2	2.159352042560013	0.3882238795413695	0.9653236587964072	1.6446773938253725	-0.3882238795413695	0.9653236587964072
**feature_2540**	DYNC1I1	1.0623550155910224	0.5129714970200236	0.9672625892747715	0.7420172427956332	-0.5129714970200236	0.9672625892747715
**feature_926**	CCND2	1.016165667087065	0.525097149885567	0.9725521913024586	0.7037689313113222	-0.525097149885567	0.9725521913024586
**feature_7988**	SLC39A10	1.0623550155910224	0.5578257328042842	0.9783512638698593	0.7190682559050466	-0.5578257328042842	0.9783512638698593
**feature_885**	PRR5	1.0623550155910224	0.46946985838343713	0.9981672383024977	0.7649662296862197	-0.46946985838343713	0.9981672383024977
**feature_1471**	SAT1	1.0392603413390438	0.8483896567823112	0.9981672383024977	0.5737246722646648	-0.8483896567823112	0.9981672383024977
**feature_2412**	PFKP	2.517319493465684	0.39176603567760204	0.9981672383024977	1.9124155742155495	-0.39176603567760204	0.9981672383024977
**feature_2860**	TNS3	2.0785206826780875	0.3948814596110808	0.9981672383024977	1.5758304331536128	-0.3948814596110808	0.9981672383024977
**feature_3032**	TRIO	4.8614289300415265	0.32948214109522844	0.9981672383024977	3.8554297976185476	-0.32948214109522844	0.9981672383024977
**feature_3927**	MAP4K3	1.1547337125989374	0.41384399861425347	0.9981672383024977	0.8644118395454283	-0.41384399861425347	0.9981672383024977
**feature_4641**	KHDRBS2	1.0854496898430013	0.5885225300272008	0.9981672383024977	0.7190682559050466	-0.5885225300272008	0.9981672383024977
**feature_5380**	NR3C2	1.9630473114181937	0.4985804047144021	0.9981672383024977	1.3845888757320577	-0.4985804047144021	0.9981672383024977
**feature_5383**	LRBA	1.3163964323627888	0.41164476794312144	0.9981672383024977	0.9868064362952235	-0.41164476794312144	0.9981672383024977
**feature_7321**	LRRC4C	2.378751447953811	0.44911947336178415	0.9981672383024977	1.736473341387719	-0.44911947336178415	0.9981672383024977
**feature_9354**	TUT4	1.0392603413390438	0.4098166430426783	0.9981672383024977	0.7802655542799442	-0.4098166430426783	0.9981672383024977
**feature_15908**	PTBP1	1.0046183299610756	0.4629916479433417	0.9981672383024977	0.7267179182019088	-0.4629916479433417	0.9981672383024977
**feature_130**	CACNA2D1	3.14087569826911	0.12285895767838872	1	2.8762730236201866	-0.12285895767838872	1
**feature_166**	ATXN7L1	1.2240177353548738	0.32998951642834745	1	0.9715071117014991	-0.32998951642834745	1
**feature_199**	TBC1D22A	1.3741331179927356	0.29574078079935495	1	1.1168506953418809	-0.29574078079935495	1
**feature_282**	PDZRN4	1.2355650724808631	0.5232310697754814	1	0.8567621772485662	-0.5232310697754814	1
**feature_301**	SCAF11	1.3394911066147674	0.09256503536478933	1	1.2545446166854004	-0.09256503536478933	1
**feature_315**	USP15	1.2124703982288845	0.06851547114681403	1	1.155099006826192	-0.06851547114681403	1
**feature_362**	ZFC3H1	2.0438786713001194	0.2633560610732937	1	1.6982250299034078	-0.2633560610732937	1
**feature_526**	ATF7IP	1.0969970269689906	0.3279949798039814	1	0.8720615018422906	-0.3279949798039814	1
**feature_655**	RBFOX2	2.0438786713001194	0.0035600293060444876	1	2.0348101709653448	-0.003560029306044487	1
**feature_773**	WNK1	1.4318698036226825	0.054460927606082066	1	1.3769392134351957	-0.054460927606082066	1
**feature_775**	ERC1	2.101615356930066	0.3493348661477744	1	1.6446773938253725	-0.3493348661477744	1
**feature_792**	MICAL3	1.8591212772842893	0.24889243340637934	1	1.5605311085598883	-0.24889243340637934	1
**feature_820**	PLEKHA5	3.4411064635448336	0.20889785923911375	1	2.9680689711825328	-0.20889785923911375	1
**feature_992**	ENSGALG00000041564	1.0623550155910224	0.26970034600765985	1	0.8797111641391527	-0.26970034600765985	1
**feature_1175**	CMSS1	2.2286360653159494	0.08514756380230626	1	2.096007469340242	-0.08514756380230626	1
**feature_1201**	ALCAM	2.3672041108278217	0.751461101153406	1	1.3998882003257822	-0.751461101153406	1
**feature_1202**	CBLB	1.1547337125989374	0.3887530176514231	1	0.8797111641391527	-0.3887530176514231	1
**feature_1231**	EPHA3	2.1708993796860026	0.37597789464709974	1	1.667626380715959	-0.37597789464709974	1
**feature_1330**	SCAF4	1.0854496898430013	0.39019381358122224	1	0.8261635280611174	-0.39019381358122224	1
**feature_1337**	PAXBP1	1.939952637166215	0.05866462874649858	1	1.858867938137514	-0.05866462874649858	1
**feature_1348**	ENSGALG00000052354	4.376440770749973	0.029095121413182046	1	4.2761612239459685	-0.029095121413182046	1
**feature_1463**	POLA1	2.9330236300013013	0.1862711857042001	1	2.5702865317456984	-0.1862711857042001	1
**feature_1485**	SH3KBP1	1.6512692090164807	0.1363957120131366	1	1.4993338101849907	-0.1363957120131366	1
**feature_1530**	MID1	2.147804705434024	0.4074965353516381	1	1.6140787446379237	-0.4074965353516381	1
**feature_1535**	TBL1X	5.381059100711049	0.006652624361425996	1	5.339464283209814	-0.006652624361425996	1
**feature_1584**	UBE3A	1.1893757239769056	0.20149940691795334	1	1.0327044100763967	-0.20149940691795334	1
**feature_1723**	ARGLU1	4.110852016852218	0.07961810728413443	1	3.878378784509134	-0.07961810728413443	1
**feature_1744**	PCCA	1.5588905120085657	0.039360750733891	1	1.5146331347787152	-0.039360750733891	1
**feature_1775**	ENSGALG00000049165	1.143186375472948	0.23237871980192537	1	0.9715071117014991	-0.23237871980192537	1
**feature_1793**	RBM26	1.8360266030323107	0.3330217968788143	1	1.4534358364038176	-0.3330217968788143	1
**feature_1800**	MYCBP2	2.621245527599588	0.09498305706963928	1	2.447891934995903	-0.09498305706963928	1
**feature_1811**	KLF12	3.1986123838990568	0.06360741297598316	1	3.052215256448017	-0.06360741297598316	1
**feature_1834**	DIAPH3	1.5473431748825763	0.3254881241934066	1	1.2315956297948139	-0.3254881241934066	1
**feature_1859**	ENSGALG00000016971	1.0392603413390438	0.008854328975557262	1	1.0327044100763967	-0.008854328975557262	1
**feature_2050**	YAP1	1.6050798605125232	0.2583739283348696	1	1.3386909019508846	-0.2583739283348696	1
**feature_2098**	TMEM135	1.5357958377565868	0.21998349284824836	1	1.315741915060298	-0.21998349284824836	1
**feature_2138**	RSF1	1.0969970269689906	0.15111721771990216	1	0.9868064362952235	-0.15111721771990216	1
**feature_2188**	RAB6A	1.29330175811081	0.3863336794139338	1	0.9868064362952235	-0.3863336794139338	1
**feature_2190**	FAM168A	1.8244792659063214	0.21490495812833765	1	1.5681807708567506	-0.21490495812833765	1
**feature_2391**	RBM33	2.0785206826780875	0.022279226652633488	1	2.042459833262207	-0.022279226652633488	1
**feature_2406**	DIP2C	6.0277099797664535	0.23117238092900738	1	5.11762407660081	-0.23117238092900738	1
**feature_2421**	NRP1	1.2471124096068524	0.206959735299445	1	1.0786023838575698	-0.206959735299445	1
**feature_2429**	SVIL	1.8475739401583	0.19834021943463243	1	1.6064290823410614	-0.19834021943463243	1
**feature_2457**	KIAA1217	1.3972277922447145	0.04352643662368905	1	1.353990226544609	-0.04352643662368905	1
**feature_2460**	PIP4K2A	1.0277130042130544	0.3661301086907528	1	0.7955648788736686	-0.3661301086907528	1
**feature_2512**	CDK14	2.58660351622162	0.33084135868761333	1	2.050109495559069	-0.33084135868761333	1
**feature_2514**	AKAP9	3.383369777914887	0.20699429293860794	1	2.9221709974013597	-0.20699429293860794	1
**feature_2525**	CDK6	1.7205532317724168	0.2704467606354447	1	1.4228371872163688	-0.2704467606354447	1
**feature_2568**	DGKB	1.8937632886622575	0.22692428970981715	1	1.6140787446379237	-0.22692428970981715	1
**feature_2581**	ENSGALG00000010854	1.143186375472948	0.08263160029724335	1	1.0786023838575698	-0.08263160029724335	1
**feature_2609**	TRA2A	1.5588905120085657	0.0249336800986113	1	1.5299324593724395	-0.0249336800986113	1
**feature_2620**	HNRNPA2B1	1.8244792659063214	0.10698961896144998	1	1.6905753676065456	-0.10698961896144998	1
**feature_2621**	CBX3	1.1200917012209695	0.16980937260495857	1	0.9944560985920857	-0.16980937260495857	1
**feature_2628**	HOXA5	1.1547337125989374	0.29253770239211996	1	0.9409084625140504	-0.29253770239211996	1
**feature_2649**	ANKRD28	1.258659746732842	0.2613577867736912	1	1.048003734670121	-0.2613577867736912	1
**feature_2680**	SLC4A7	1.8937632886622575	0.16694218488158463	1	1.6829257053096836	-0.16694218488158463	1
**feature_2781**	CLASP2	1.7090058946464275	0.18563923103298585	1	1.4993338101849907	-0.18563923103298585	1
**feature_2829**	ENSGALG00000043243	1.0277130042130544	0.23488557541250016	1	0.8720615018422906	-0.23488557541250016	1
**feature_2852**	CUL1	1.3279437694887781	0.1427227769776696	1	1.200996980607365	-0.1427227769776696	1
**feature_2900**	CDKAL1	3.6489585318126427	0.24580986898767931	1	3.0675145810417415	-0.24580986898767931	1

**feature_2934**	HIVEP1	2.2055413910639707	0.1904914560540466	1	1.9277148988092738	-0.1904914560540466	1
**feature_2957**	RREB1	1.47805915212664	0.20743236267169457	1	1.277493603575987	-0.20743236267169457	1
**feature_2967**	PRPF4B	1.9976893227961618	0.30354557131272986	1	1.6140787446379237	-0.30354557131272986	1
**feature_3149**	ZNF516	1.8013845916543425	0.3285544272411902	1	1.430486849513231	-0.3285544272411902	1
**feature_3234**	SMCHD1	1.3625857808667463	0.10830086198821948	1	1.2621942789822627	-0.10830086198821948	1
**feature_3249**	MIB1	1.47805915212664	0.1485386736181259	1	1.3310412396540223	-0.1485386736181259	1
**feature_3288**	ASXL3	1.3048490952367995	0.2818611917931488	1	1.0709527215607078	-0.2818611917931488	1
**feature_3302**	SNTG1	1.0046183299610756	0.24059922660689365	1	0.8491125149517039	-0.24059922660689365	1
**feature_3325**	PLAG1	1.0277130042130544	0.16151238908228077	1	0.9179594756234637	-0.16151238908228077	1
**feature_3339**	CHD7	3.0946863497651527	0.22119304000556372	1	2.6467831547143206	-0.22119304000556372	1
**feature_3384**	KCNB2	1.5588905120085657	0.34506849316292293	1	1.2239459674979516	-0.34506849316292293	1
**feature_3533**	ZFPM2	1.5935325233865338	0.013976645251382869	1	1.5758304331536128	-0.013976645251382869	1
**feature_3617**	ASAP1	1.7898372545283532	0.14600429432624973	1	1.6140787446379237	-0.14600429432624973	1
**feature_3627**	PHF20L1	1.7205532317724168	0.049156895746155116	1	1.659976718419097	-0.049156895746155116	1
**feature_3710**	CCDC88A	1.1316390383469588	0.32306138679886676	1	0.9026601510297393	-0.32306138679886676	1
**feature_3731**	RTN4	1.755195243150385	0.025731410304664637	1	1.7211740167939944	-0.025731410304664637	1
**feature_3735**	PSME4	1.016165667087065	0.30936845883012987	1	0.8185138657642551	-0.30936845883012987	1
**feature_3793**	NRXN1	1.6397218718904913	0.33054135191969225	1	1.3004425904665735	-0.33054135191969225	1
**feature_3824**	EHBP1	1.3741331179927356	0.26659443513983816	1	1.1397996822324674	-0.26659443513983816	1
**feature_3916**	HNRNPLL	1.3163964323627888	0.08528442674049835	1	1.239245292091676	-0.08528442674049835	1
**feature_3946**	LBR	1.3741331179927356	0.23802528294306732	1	1.162748669123054	-0.23802528294306732	1
**feature_3961**	MIA3	1.0854496898430013	0.22764080079526217	1	0.925609137920326	-0.22764080079526217	1
**feature_3979**	GPATCH2	1.3972277922447145	0.06804875575775793	1	1.3310412396540223	-0.06804875575775793	1
**feature_4031**	LRPPRC	1.7090058946464275	0.039506725712435964	1	1.659976718419097	-0.039506725712435964	1
**feature_4047**	TTC7A	1.0392603413390438	0.3281328454489704	1	0.8261635280611174	-0.3281328454489704	1
**feature_4104**	LTBP1	2.424940796457769	0.07600204197468341	1	2.2948986890586593	-0.07600204197468341	1
**feature_4140**	HNRNPU	1.7782899174023639	0.35685229510274175	1	1.3845888757320577	-0.35685229510274175	1
**feature_4147**	CEP170	2.124710031182045	0.2557178458892325	1	1.7747216528720298	-0.2557178458892325	1
**feature_4264**	WTAP	1.2240177353548738	0.16006451498603516	1	1.0939017084512943	-0.16006451498603516	1
**feature_4269**	UTRN	3.221707058151036	0.37350963905635015	1	2.478490584183352	-0.37350963905635015	1
**feature_4294**	MTHFD1L	1.1778283868509163	0.1769909742498922	1	1.040354072373259	-0.1769909742498922	1
**feature_4364**	BCLAF1	1.9284053000402257	0.08602218949801743	1	1.8129699643563408	-0.08602218949801743	1
**feature_4432**	CEP85L	1.258659746732842	0.07526149466023319	1	1.1933473183105028	-0.07526149466023319	1
**feature_4501**	SOBP	2.1131626940560557	0.3307247725595538	1	1.6752760430128213	-0.3307247725595538	1
**feature_4547**	MDN1	1.016165667087065	0.30936845883012987	1	0.8185138657642551	-0.30936845883012987	1
**feature_4557**	RNGTT	1.1893757239769056	0.04103473472470717	1	1.155099006826192	-0.04103473472470717	1
**feature_4593**	PHIP	3.1177810240171313	0.005335654195895767	1	3.09811323022919	-0.005335654195895767	1
**feature_4638**	PHF3	1.143186375472948	0.08263160029724335	1	1.0786023838575698	-0.08263160029724335	1
**feature_4676**	MCPH1	1.1200917012209695	0.03330737270009651	1	1.0939017084512943	-0.03330737270009651	1
**feature_4733**	ROCK2	1.4203224664966931	0.23893809223697288	1	1.200996980607365	-0.23893809223697288	1
**feature_4764**	PUM2	1.755195243150385	0.07772604794292615	1	1.659976718419097	-0.07772604794292615	1
**feature_5114**	ENSGALG00000006851	1.1316390383469588	0.17348503074835672	1	1.0021057608889479	-0.17348503074835672	1
**feature_5223**	STAG2	1.166281049724927	0.24972061657544203	1	0.9791567739983613	-0.24972061657544203	1
**feature_5351**	ENSGALG00000009809	3.972283971340345	0.18925234660667334	1	3.472946682775438	-0.18925234660667334	1
**feature_5357**	RNF150	1.050807678465033	0.1120844860842134	1	0.9715071117014991	-0.1120844860842134	1
**feature_5372**	SLC10A7	1.200923061102895	0.017365828831074903	1	1.1856976560136407	-0.017365828831074903	1
**feature_5604**	ADAMTS3	1.8129319287803318	0.7123941881893692	1	1.1015513707481566	-0.7123941881893692	1
**feature_5612**	EPHA5	1.351038443740757	0.4487977663605874	1	0.9868064362952235	-0.4487977663605874	1
**feature_5687**	RAP1GDS1	1.8475739401583	0.13153684875038585	1	1.6829257053096836	-0.13153684875038585	1
**feature_5705**	PPP3CA	1.143186375472948	0.042554160921908224	1	1.1092010330450186	-0.042554160921908224	1
**feature_5726**	FAT1	1.1778283868509163	0.15608198448691152	1	1.0556533969669832	-0.15608198448691152	1
**feature_5855**	DHX15	1.0854496898430013	0.3381991759429608	1	0.8567621772485662	-0.3381991759429608	1
**feature_5863**	SLIT2	5.4965324719709425	0.2965434271526952	1	4.459753119070661	-0.2965434271526952	1
**feature_5911**	KIAA0232	1.6397218718904913	0.05490690930626474	1	1.5758304331536128	-0.05490690930626474	1
**feature_5966**	CTBP1	3.4642011377968127	0.22595245064249214	1	2.9527696465888082	-0.22595245064249214	1
**feature_6016**	CTNNA2	6.062351991144422	0.18657235170863284	1	5.308865634022365	-0.18657235170863284	1
**feature_6343**	ENSGALG00000014734	1.3741331179927356	0.0196796946693214	1	1.353990226544609	-0.0196796946693214	1
**feature_6344**	KIF2A	1.0277130042130544	0.2990159128322157	1	0.8338131903579795	-0.2990159128322157	1
**feature_6546**	DAPK1	1.258659746732842	0.3254881241934067	1	1.0021057608889479	-0.3254881241934067	1
**feature_6584**	ROR2	3.2448017324030145	0.06273796524081776	1	3.09811323022919	-0.06273796524081776	1
**feature_6597**	MCC	1.9168579629142362	0.10804067784697269	1	1.7747216528720298	-0.10804067784697269	1
**feature_6729**	MSH3	1.016165667087065	0.05302870557034428	1	0.9791567739983613	-0.05302870557034428	1
**feature_6798**	DMXL1	1.050807678465033	0.1348045625842969	1	0.9562077871077748	-0.1348045625842969	1
**feature_6813**	ENSGALG00000002439	1.0392603413390438	0.3281328454489704	1	0.8261635280611174	-0.3281328454489704	1
**feature_6885**	ZNF608	3.9607366342143555	0.29711086166290257	1	3.212858164682123	-0.29711086166290257	1
**feature_7097**	SPON1	1.47805915212664	0.41984069543552044	1	1.1015513707481566	-0.41984069543552044	1
**feature_7152**	SOX6	1.47805915212664	0.36134504612537577	1	1.1474493445293297	-0.36134504612537577	1
**feature_7217**	TSPAN4	1.7782899174023639	0.1164540857119778	1	1.6370277315285102	-0.1164540857119778	1
**feature_7299**	CAPRIN1	2.0438786713001194	0.09847167633151144	1	1.9047659119186873	-0.09847167633151144	1
**feature_7343**	CELF1	1.258659746732842	0.10309570285695882	1	1.1703983314199162	-0.10309570285695882	1
**feature_7358**	CKAP5	1.143186375472948	0.072507383023536	1	1.086252046154432	-0.072507383023536	1
**feature_7383**	INO80	1.5127011635046081	0.3475144305234056	1	1.1856976560136407	-0.3475144305234056	1
**feature_7437**	ENSGALG00000047486	1.7782899174023639	0.03809844801052298	1	1.7288236790908567	-0.03809844801052298	1
**feature_7443**	PCNX1	1.3972277922447145	0.3000913471279254	1	1.1321500199356052	-0.3000913471279254	1
**feature_7452**	SMOC1	2.7829082473634394	0.38326445547260746	1	2.126606118527691	-0.38326445547260746	1
**feature_7465**	RAD51B	2.621245527599588	0.029095121413182046	1	2.5626368694488364	-0.029095121413182046	1
**feature_7508**	NOVA1	5.288680403703133	0.31713070739182037	1	4.230263250164795	-0.31713070739182037	1
**feature_7554**	PNN	3.14087569826911	0.14600429432624984	1	2.830375049839013	-0.14600429432624984	1
**feature_7642**	CCDC88C	1.3048490952367995	0.29212952724697583	1	1.0633030592638455	-0.29212952724697583	1
**feature_7717**	CDC42BPB	1.6743638832684593	0.1059217469357221	1	1.552881446263026	-0.1059217469357221	1
**feature_7723**	MARK3	1.6974585575204382	0.2510814357685137	1	1.4228371872163688	-0.2510814357685137	1
**feature_7761**	SYNE2	1.9514999742922043	0.0970466581841537	1	1.820619626653203	-0.0970466581841537	1
**feature_7945**	AGAP1	2.4595828078357367	0.1751714073762436	1	2.172504092308864	-0.1751714073762436	1
**feature_8056**	PARD3B	3.6143165204346746	0.35566627285843166	1	2.815075725245289	-0.35566627285843166	1
**feature_8136**	TLK1	1.8475739401583	0.2612279253900227	1	1.5375821216693017	-0.2612279253900227	1
**feature_8270**	CLASP1	1.5127011635046081	0.018206805270445714	1	1.4916841478881286	-0.018206805270445714	1
**feature_8279**	SEMA5B	2.1362573683080344	0.24503829177339087	1	1.7976706397626165	-0.24503829177339087	1
**feature_8282**	ADCY6	1.3741331179927356	0.21001090677347078	1	1.1856976560136407	-0.21001090677347078	1
**feature_8324**	NCKAP5	1.7321005688984064	0.07873441976281914	1	1.6370277315285102	-0.07873441976281914	1
**feature_8335**	R3HDM1	1.0277130042130544	0.14963828879398905	1	0.925609137920326	-0.14963828879398905	1
**feature_8345**	SPOPL	1.1316390383469588	0.3977001876916225	1	0.8567621772485662	-0.3977001876916225	1
**feature_8414**	WAPL	1.1316390383469588	0.34751443052340575	1	0.8873608264360149	-0.34751443052340575	1
**feature_8518**	HNRNPH3	2.066973345552098	0.08037562635687545	1	1.9506638856998604	-0.08037562635687545	1
**feature_8524**	ENSGALG00000004108	1.1778283868509163	0.24160005567296872	1	0.9944560985920857	-0.24160005567296872	1
**feature_8576**	DLG5	1.0046183299610756	0.30648716226335093	1	0.810864203467393	-0.30648716226335093	1
**feature_8588**	ADK	2.009236659922151	0.09721913652028946	1	1.8741672627312385	-0.09721913652028946	1
**feature_8602**	PPP3CB	1.5011538263786188	0.15433998148165323	1	1.3463405642477468	-0.15433998148165323	1
**feature_8764**	BTRC	1.0854496898430013	0.288013418774744	1	0.8873608264360149	-0.288013418774744	1
**feature_8809**	SORCS1	1.385680455118725	0.06444227145306658	1	1.3233915773571603	-0.06444227145306658	1
**feature_8826**	VTI1A	1.4549644778746613	0.15935470191346868	1	1.3004425904665735	-0.15935470191346868	1
**feature_8843**	ATRNL1	3.5334851605527486	0.18173239113113304	1	3.105762892526052	-0.18173239113113304	1
**feature_9038**	PRRC2C	2.4826774820877158	0.012493154076052004	1	2.4555415972927657	-0.012493154076052004	1
**feature_9090**	RASAL2	1.3279437694887781	0.09777537092369504	1	1.239245292091676	-0.09777537092369504	1
**feature_9133**	IVNS1ABP	1.050807678465033	0.003560029306044321	1	1.048003734670121	-0.003560029306044321	1
**feature_9137**	RNPC3	1.5357958377565868	0.01798709994132419	1	1.5146331347787152	-0.01798709994132419	1
**feature_9165**	DPYD	2.7482662359854713	0.1318373333898657	1	2.5014395710739388	-0.1318373333898657	1
**feature_9208**	PKN2	1.016165667087065	0.12174145565435844	1	0.9332588002171881	-0.12174145565435844	1
**feature_9252**	ZZZ3	1.0046183299610756	0.09375783827762256	1	0.9409084625140504	-0.09375783827762256	1
**feature_9271**	ST3GAL3	1.0277130042130544	0.2100109067734709	1	0.8873608264360149	-0.2100109067734709	1
**feature_9304**	NASP	2.759813573111461	0.03188875236453903	1	2.6926811284954937	-0.03188875236453903	1
**feature_9337**	BEND5	2.344109436575843	0.44076890958350823	1	1.7211740167939944	-0.44076890958350823	1
**feature_9340**	FAF1	1.073902352717012	0.2727466621214335	1	0.8873608264360149	-0.2727466621214335	1
**feature_9512**	GPC1	1.385680455118725	0.19450281201743963	1	1.2086466429042273	-0.19450281201743963	1
**feature_9711**	LPP	1.6859112203944489	0.2107660468278163	1	1.4534358364038176	-0.2107660468278163	1
**feature_9792**	FXR1	1.1200917012209695	0.24903606375553355	1	0.9409084625140504	-0.24903606375553355	1
**feature_9809**	NAALADL2	1.10854436409498	0.1331529367131385	1	1.0097554231858101	-0.1331529367131385	1
**feature_9810**	NLGN1	1.2124703982288845	0.3739284792511597	1	0.9332588002171881	-0.3739284792511597	1
**feature_9836**	ENSGALG00000009458	1.6166271976385125	0.2362207860963193	1	1.3692895511383334	-0.2362207860963193	1
**feature_10067**	TJP1	2.575056179095631	0.21100210917652085	1	2.218402066090037	-0.21100210917652085	1
**feature_10094**	NEDD4	1.5011538263786188	0.3273247826730237	1	1.1933473183105028	-0.3273247826730237	1
**feature_10265**	AAGAB	1.4665118150006506	0.2576056519125836	1	1.2239459674979516	-0.2576056519125836	1
**feature_10347**	GNL3	1.1547337125989374	0.20233989342054168	1	1.0021057608889479	-0.20233989342054168	1
**feature_10422**	RBM10	3.0600443383871845	0.058842464807234096	1	2.9298206596982217	-0.058842464807234096	1
**feature_10441**	IARS1	1.200923061102895	0.19424359091515425	1	1.048003734670121	-0.19424359091515425	1
**feature_10604**	MAGI1	2.852192270119376	0.007443463793272542	1	2.830375049839013	-0.007443463793272542	1
**feature_10650**	SRGAP3	1.385680455118725	0.08112101259969717	1	1.3080922527634358	-0.08112101259969717	1
**feature_10833**	ZNF423	5.334869752207092	0.13277813162790864	1	4.849885896210633	-0.13277813162790864	1
**feature_10911**	CDH11	1.2124703982288845	0.28301998305294945	1	0.9944560985920857	-0.28301998305294945	1
**feature_10948**	USP10	1.2471124096068524	0.30142441930293734	1	1.0097554231858101	-0.30142441930293734	1

**feature_11021**	NFAT5	1.5242485006305975	0.1124235050120781	1	1.4075378626226445	-0.1124235050120781	1
**feature_11131**	CTNNA1	1.1200917012209695	0.3452513790148367	1	0.8797111641391527	-0.3452513790148367	1
**feature_11137**	NPM1	1.4434171407486718	0.1395376988250191	1	1.3080922527634358	-0.1395376988250191	1
**feature_11157**	HMMR	1.0854496898430013	0.1249551368206983	1	0.9944560985920857	-0.1249551368206983	1
**feature_11322**	HNRNPH2	3.1177810240171313	0.10081596305012419	1	2.899222010510773	-0.10081596305012419	1
**feature_11413**	TCERG1	1.4665118150006506	0.12091697994420308	1	1.3463405642477468	-0.12091697994420308	1
**feature_11487**	MAD1L1	2.6905295503555244	0.3503627928324362	1	2.1036571316371044	-0.3503627928324362	1
**feature_11503**	SDK1	2.621245527599588	0.21195917856299262	1	2.2566503775743483	-0.21195917856299262	1
**feature_11531**	USP22	1.7667425802763743	0.26285203345402164	1	1.4687351609975419	-0.26285203345402164	1
**feature_11648**	XYLT1	1.4318698036226825	0.01514800358125612	1	1.4151875249195065	-0.01514800358125612	1
**feature_11717**	LUC7L	2.4480354707097476	0.09441345975715776	1	2.2872490267617973	-0.09441345975715776	1
**feature_11806**	MAPK8IP3	1.5588905120085657	0.12909091138990325	1	1.4228371872163688	-0.12909091138990325	1
**feature_11848**	DLGAP4	1.1778283868509163	0.24160005567296872	1	0.9944560985920857	-0.24160005567296872	1
**feature_11863**	PHF20	1.5473431748825763	0.007126644326236553	1	1.5375821216693017	-0.007126644326236553	1
**feature_12197**	MAPK3	1.385680455118725	0.39886131052362594	1	1.048003734670121	-0.39886131052362594	1
**feature_12224**	ENSGALG00000054877	1.4318698036226825	0.10309570285695857	1	1.3310412396540223	-0.10309570285695857	1
**feature_12231**	SFSWAP	1.166281049724927	0.27226318522653026	1	0.9638574494046369	-0.27226318522653026	1
**feature_12307**	RSRC2	1.6859112203944489	0.11576953289206926	1	1.552881446263026	-0.11576953289206926	1
**feature_12493**	CIT	1.0392603413390438	0.06289416868844648	1	0.9944560985920857	-0.06289416868844648	1
**feature_12680**	CSNK1D	1.5935325233865338	0.049252119998913324	1	1.5375821216693017	-0.049252119998913324	1
**feature_12727**	DDX5	5.53117448334891	0.18480034397619072	1	4.849885896210633	-0.18480034397619072	1
**feature_12745**	CEP112	1.0277130042130544	0.2474856121921337	1	0.8644118395454283	-0.2474856121921337	1
**feature_12967**	EHMT1	1.8129319287803318	0.26294665812469376	1	1.506983472481853	-0.26294665812469376	1
**feature_12988**	ENSGALG00000048936	1.016165667087065	0.14539272371900377	1	0.9179594756234637	-0.14539272371900377	1
**feature_13104**	VAV2	1.0623550155910224	0.1969440035723456	1	0.925609137920326	-0.1969440035723456	1
**feature_13163**	CRB2	1.351038443740757	0.31129424261065247	1	1.086252046154432	-0.31129424261065247	1
**feature_13164**	DENND1A	2.5404141677176626	0.1669717237896824	1	2.2566503775743483	-0.1669717237896824	1
**feature_13199**	ENSGALG00000037051	1.0046183299610756	0.025586335636043345	1	0.9868064362952235	-0.025586335636043345	1
**feature_13203**	ENSGALG00000054915	4.260967399490079	0.10867673512025866	1	3.939576082884032	-0.10867673512025866	1
**feature_13218**	BAZ1B	2.494224819213705	0.023644275349376853	1	2.447891934995903	-0.023644275349376853	1
**feature_13487**	MSI2	3.9491892970883664	0.059302581233040996	1	3.7789331746499255	-0.059302581233040996	1
**feature_13489**	NF1	1.8475739401583	0.15788817569733826	1	1.6523270561222347	-0.15788817569733826	1
**feature_13802**	TOP2A	1.4896064892526293	0.07950888253934285	1	1.4075378626226445	-0.07950888253934285	1
**feature_14349**	ENSGALG00000002232	2.286372750945896	0.12711506761032276	1	2.08835780704338	-0.12711506761032276	1
**feature_14365**	KIF1B	1.5357958377565868	0.23675920559907537	1	1.3004425904665735	-0.23675920559907537	1
**feature_14492**	EIF4G3	1.4318698036226825	0.32548812419340645	1	1.1397996822324674	-0.32548812419340645	1
**feature_14503**	ENSGALG00000027170	3.3487277665369186	0.37679309175362596	1	2.5702865317456984	-0.37679309175362596	1
**feature_14524**	HINTW	5.184754369569229	0.1192153682202357	1	4.758089948648287	-0.1192153682202357	1
**feature_14562**	ENSGALG00000030382	1.1778283868509163	0.35615981993582435	1	0.9179594756234637	-0.35615981993582435	1
**feature_14600**	C2CD2L	1.3048490952367995	0.3553233537341698	1	1.0174050854826724	-0.3553233537341698	1
**feature_15130**	RSRP1	1.4549644778746613	0.11777559816206906	1	1.3386909019508846	-0.11777559816206906	1
**feature_15180**	THRAP3	1.8013845916543425	0.34398446781079617	1	1.4151875249195065	-0.34398446781079617	1
**feature_15270**	SRSF10	2.621245527599588	0.07711663627612553	1	2.478490584183352	-0.07711663627612553	1
**feature_15489**	CSDE1	1.3048490952367995	0.08156254130731755	1	1.2315956297948139	-0.08156254130731755	1
**feature_15765**	TIMM44	1.143186375472948	0.3254881241934069	1	0.9103098133266015	-0.3254881241934069	1
**feature_15829**	ENSGALG00000001948	1.0277130042130544	0.2990159128322157	1	0.8338131903579795	-0.2990159128322157	1
**feature_15871**	REXO1	1.200923061102895	0.3369837630312362	1	0.9485581248109125	-0.3369837630312362	1
**feature_16039**	KDM4B	1.3279437694887781	0.17037911988053495	1	1.1780479937167785	-0.17037911988053495	1

List of MEK1ca versus control DGEs among GFP-positive cells – Downregulated genes:

FeatureID	FeatureName	GFP+sup1 MEK1ca Average	GFP+sup1 MEK1ca Log2 Fold Change	GFP+sup1 MEK1ca P-Value	GFP+sup1 Control Average	GFP+sup1 Control Log2 Fold Change	GFP+sup1 Control P-Value
**feature_8353**	ZEB2	4.318704085120026	-1.5045868743642807	0.004102573110260808	12.231810012682654	1.5045868743642807	0.004102573110260808
**feature_6613**	EFNA5	2.5981508533476094	-1.441485345203386	0.007678709730499681	7.052988637706946	1.441485345203386	0.007678709730499681
**feature_4420**	CLVS2	0.20785206826780875	-2.2079440758876676	0.00918772359299095	1.0021057608889479	2.2079440758876676	0.00918772359299095
**feature_5667**	ENSGALG00000012068	0.2309467425197875	-2.3045622660562874	0.011980373031710505	1.1856976560136407	2.3045622660562874	0.011980373031710505
**feature_5873**	LDB2	1.8591212772842893	-1.398056954210478	0.012760713400225013	4.903433532288669	1.398056954210478	0.012760713400225013
**feature_3517**	NCALD	0.38106212515764937	-1.5431831491846215	0.01627113089517309	1.1321500199356052	1.5431831491846215	0.01627113089517309
**feature_5957**	FGFR3	1.6281745347645018	-1.2589098314057388	0.034143213302971244	3.9013277713997208	1.2589098314057388	0.034143213302971244
**feature_1518**	GPM6B	1.7090058946464275	-1.1348234548498777	0.0347630952266143	3.755984187759339	1.1348234548498777	0.0347630952266143
**feature_15742**	PPIA	0.7043875646853519	-1.1577208777270163	0.04177043824745938	1.5834800954504749	1.1577208777270163	0.041770438247459976
**feature_3177**	CDH7	0.7274822389373307	-1.7288900837376688	0.05024442247397461	2.4325926104021787	1.7288900837376688	0.05024442247397461
**feature_5588**	SHROOM3	0.4734408221655644	-1.5029758244582956	0.053832579548616984	1.3616398888414711	1.5029758244582956	0.053832579548616984
**feature_6787**	XPA	1.7321005688984064	-0.9145397170488676	0.054741294579044836	3.2664058007601584	0.9145397170488676	0.054741294579044836
**feature_14749**	NCAM1	1.3741331179927356	-1.035968334880593	0.06345314397273397	2.822725387542151	1.035968334880593	0.06345314397273397
**feature_3584**	ENPP2	0.6697455533073837	-1.2499336093181432	0.0965691615864051	1.6064290823410614	1.2499336093181432	0.0965691615864051
**feature_1496**	NHS	2.1362573683080344	-1.0987308516535539	0.09736527648734174	4.574498053523595	1.0987308516535539	0.09736527648734174
**feature_5447**	ENSGALG00000054138	0.33487277665369186	-2.004432761447486	0.10006175334083109	1.3769392134351957	2.004432761447486	0.10006175334083109
**feature_10592**	FHIT	0.43879881078759625	-1.465925253995176	0.10054216095939754	1.2315956297948139	1.465925253995176	0.10054216095939754
**feature_9973**	NEO1	1.8360266030323107	-1.1229723766228874	0.11704672488296944	4.000773381258929	1.1229723766228874	0.11704672488296944
**feature_4157**	ENSGALG00000031427	0.7621242503152987	-1.3621965672015808	0.11848374114559257	1.973612872590447	1.3621965672015808	0.11848374114559247
**feature_2466**	NEBL	1.385680455118725	-1.1895817589324922	0.12183394542653031	3.1669601909009497	1.1895817589324922	0.12183394542653204
**feature_6527**	TLE1	0.9468816443311288	-1.1291951795457271	0.12646446839201556	2.080708144746518	1.1291951795457271	0.12646446839201556
**feature_7206**	BRSK1	1.8360266030323107	-1.1202165113323783	0.12874516286002066	3.9931237189620674	1.1202165113323783	0.12874516286002066
**feature_836**	ST8SIA1	0.7621242503152987	-1.0868552659161739	0.1419834754161311	1.6293780692316482	1.0868552659161739	0.1419834754161311
**feature_16228**	ENSGALG00000037441	0.5658195191734794	-1.0622420290897017	0.15070558320900454	1.1933473183105028	1.0622420290897017	0.15070558320900454
**feature_10218**	NR2F2	0.7274822389373307	-1.069688952724594	0.15342340490328119	1.5375821216693017	1.069688952724594	0.15342340490327902
**feature_2817**	ITGA9	0.8660502844492032	-1.2615820394897408	0.15774866447897762	2.08835780704338	1.2615820394897408	0.15774866447897762
**feature_5365**	HHIP	0.4272514736616069	-1.2510127977795533	0.1805053285078107	1.0327044100763967	1.2510127977795533	0.1805053285078107
**feature_6643**	TRABD2A	0.15011538263786187	-2.8037948927515597	0.19492539555605223	1.1168506953418809	2.8037948927515597	0.19492539555605223
**feature_5495**	GALNTL6	0.4272514736616069	-1.4114774699727997	0.2058193595631195	1.155099006826192	1.4114774699727997	0.2058193595631195
**feature_5364**	ANAPC10	0.9930709928350863	-0.9458138976239875	0.2210957502469626	1.9200652365124116	0.9458138976239875	0.2210957502469626
**feature_95**	CELF2	6.766739555829774	-0.799223277583903	0.2293027134455628	11.742231625683473	0.799223277583903	0.2293027134455628
**feature_10065**	APBA2	0.7967662616932669	-0.8969042971430414	0.2293027134455628	1.4916841478881286	0.8969042971430414	0.2293027134455628
**feature_11586**	RPS2	0.4272514736616069	-1.2296391469869865	0.2320643993286919	1.0174050854826724	1.2296391469869865	0.2320643993286919
**feature_279**	NRCAM	1.2471124096068524	-0.9425011635831517	0.2499292675112397	2.40199396121473	0.9425011635831517	0.2499292675112397
**feature_6478**	ADAMTSL1	0.39260946228363874	-1.3696572942781724	0.2544801036795234	1.0327044100763967	1.3696572942781724	0.2544801036795234
**feature_6711**	MEF2C	0.5773668562994687	-1.8631734398304913	0.25987668938721564	2.126606118527691	1.8631734398304913	0.25987668938721564
**feature_5116**	DACH2	3.8106212515764937	-0.8368296719953516	0.2849244810084043	6.792900119613631	0.8368296719953516	0.2849244810084043
**feature_8354**	ACVR2A	0.646650879055405	-0.8453739965029591	0.2863251251422101	1.1703983314199162	0.8453739965029591	0.2863251251422101
**feature_9615**	PAX3	1.9284053000402257	-0.9410009842685292	0.3122616337491819	3.702436551681304	0.9410009842685292	0.3122616337491819
**feature_10818**	FTO	0.5080828335435325	-0.9640184930015782	0.32886190160480777	1.0021057608889479	0.9640184930015782	0.32886190160480777
**feature_2221**	HBE	0.20785206826780875	-2.5898147112313117	0.3328554528415004	1.3080922527634358	2.5898147112313117	0.3328554528415004
**feature_11707**	HBZ	0.3233254395277025	-2.7871161516049296	0.3609759971055267	2.2948986890586593	2.7871161516049296	0.3609759971055267
**feature_272**	IMMP2L	1.0854496898430013	-0.8472464108357295	0.36914816851005894	1.9583135479967226	0.8472464108357295	0.36914816851005894
**feature_835**	C2CD5	0.7274822389373307	-0.769029474590883	0.3737435260400301	1.2468949543885381	0.769029474590883	0.3737435260400301
**feature_2225**	HBBR	0.2540414167717662	-2.403615352199829	0.37570810247693676	1.39223853802892	2.403615352199829	0.37570810247693676
**feature_10216**	RGMA	0.5658195191734794	-0.9672936250344388	0.37570810247693676	1.1168506953418809	0.9672936250344388	0.37570810247693676
**feature_14440**	DHRS3	1.1778283868509163	-0.9246492876259453	0.40986532603369047	2.2413510529806238	0.9246492876259453	0.40986532603369047
**feature_6569**	SPIN1Z	0.9006922958271713	-0.7941972489789149	0.4178549443426061	1.5681807708567506	0.7941972489789149	0.4178549443426061
**feature_8287**	KALRN	1.4434171407486718	-0.8562623126456952	0.4210146578710751	2.6161845055268715	0.8562623126456952	0.4210146578710751
**feature_4440**	SLC35F1	3.4411064635448336	-0.6913015337704449	0.42104469137928774	5.546005165225093	0.6913015337704449	0.42104469137928774
**feature_1527**	FRMPD4	0.8198609359452457	-0.8709090886100967	0.42498874944346166	1.506983472481853	0.8709090886100967	0.42498874944346166
**feature_8179**	FIGN	1.2124703982288845	-0.7550193779662246	0.42498874944346166	2.050109495559069	0.7550193779662246	0.42498874944346166
**feature_1605**	AFF3	1.570437849134555	-0.8132912740954782	0.4251038200757665	2.7615280891672533	0.8132912740954782	0.4251038200757665
**feature_7499**	MEIS2	4.133946691104196	-0.6667888302622482	0.4296855589994978	6.548110926114041	0.6667888302622482	0.4296855589994978
**feature_887**	PHF21B	0.6581982161813944	-0.8388986937074758	0.4330141530815264	1.1856976560136407	0.8388986937074758	0.4330141530815264
**feature_3370**	PREX2	0.646650879055405	-0.8453739965029591	0.4440727987707245	1.1703983314199162	0.8453739965029591	0.4440727987707245
**feature_12330**	RPL6	0.6120088676774369	-0.8562623126456951	0.444591298699456	1.1168506953418809	0.8562623126456951	0.4445912986994623
**feature_5077**	GPC4	0.9584289814571181	-0.8457085344849542	0.46381636040712276	1.7288236790908567	0.8457085344849542	0.46381636040712276
**feature_5491**	TENM3	2.6443402018515667	-0.7029785311086536	0.4653066258967299	4.299110210836555	0.7029785311086536	0.4653066258967299
**feature_1176**	ENSGALG00000053198	1.1778283868509163	-0.7306014919834591	0.47842209770860905	1.9583135479967226	0.7306014919834591	0.47842209770860905
**feature_3219**	LAMA1	1.10854436409498	-0.7124314357664203	0.47842209770860905	1.820619626653203	0.7124314357664203	0.47842209770860905
**feature_11431**	SHISA9	1.5011538263786188	-1.0182994046576952	0.47842209770860905	3.044565594151155	1.0182994046576952	0.47842209770860905
**feature_15927**	RBM3	0.9468816443311288	-0.7368777567669669	0.47842209770860905	1.5834800954504749	0.7368777567669669	0.47842209770860905
**feature_4541**	EPHA7	1.5242485006305975	-0.6960450373562346	0.4859570655407906	2.4708409218864897	0.6960450373562346	0.4859570655407906
**feature_6476**	CNTLN	0.6697455533073837	-0.8686844234955828	0.4859570655407906	1.2315956297948139	0.8686844234955828	0.4859570655407906
**feature_7707**	HSP90AA1	1.0277130042130544	-0.7075269330345563	0.48992582059047507	1.6829257053096836	0.7075269330345563	0.48992582059047507
**feature_6724**	SSBP2	1.0046183299610756	-0.7399484107269338	0.4938492750365357	1.6829257053096836	0.7399484107269338	0.4938492750365357
**feature_15596**	EBF2	0.38106212515764937	-1.4635659811212536	0.5060555818069258	1.0709527215607078	1.4635659811212536	0.5060555818069258
**feature_9334**	TRABD2B	0.6004615305514475	-1.0144738935242175	0.5130907023864442	1.2239459674979516	1.0144738935242175	0.5130907023864442
**feature_13116**	NOTCH1	0.6120088676774369	-0.7755310405328383	0.5263268374127504	1.0556533969669832	0.7755310405328383	0.5263268374127504
**feature_2673**	RARB	2.1131626940560557	-0.6055919369821825	0.5537450511665621	3.212858164682123	0.6055919369821825	0.5537450511665621
**feature_7907**	MAP2	0.6581982161813944	-0.9713489897311252	0.5553590888131095	1.3004425904665735	0.9713489897311252	0.5553590888131095

**feature_10859**	ZNF536	0.6351035419294156	-0.9260506428025577	0.5553590888131095	1.2162963052010893	0.9260506428025577	0.5553590888131095
**feature_7814**	PELI2	0.7736715874412882	-0.8709090886100967	0.5607815075032229	1.4228371872163688	0.8709090886100967	0.5607815075032229
**feature_10629**	PDZRN3	0.7043875646853519	-0.7610619077630272	0.5727717201370638	1.200996980607365	0.7610619077630272	0.5727717201370638
**feature_16835**	RPS19	0.55427218204749	-0.9052920826357603	0.573185194889362	1.048003734670121	0.9052920826357603	0.573185194889362
**feature_3651**	TSNARE1	1.4549644778746613	-0.6273639274498372	0.5903609566381205	2.2490007152774862	0.6273639274498372	0.5903609566381205
**feature_1974**	RPL21	0.6928402275593625	-0.6999086528720746	0.6028661388047021	1.1321500199356052	0.6999086528720746	0.6028661388047021
**feature_8593**	ENSGALG00000005107	0.7043875646853519	-0.7426833784481726	0.6028661388047021	1.1856976560136407	0.7426833784481726	0.6028661388047021
**feature_9653**	TFDP2	0.6928402275593625	-0.7568645376841507	0.6035984118238852	1.1780479937167785	0.7568645376841507	0.6035984118238852
**feature_12163**	ADNP	1.4434171407486718	-0.5737488988716766	0.6103363342246665	2.1495551054182775	0.5737488988716766	0.6103363342246492
**feature_6403**	ENSGALG00000051796	1.2471124096068524	-0.603295077263954	0.6466091532170549	1.897116249621825	0.603295077263954	0.6466091532170549
**feature_2468**	PLXDC2	1.2240177353548738	-0.8745816278208561	0.6560308759974934	2.2490007152774862	0.8745816278208561	0.6560308759974934
**feature_4936**	ENSGALG00000029012	0.6235562048034262	-0.7490588291716471	0.6560308759974934	1.0556533969669832	0.7490588291716471	0.6560308759974934
**feature_11708**	HBAD	0.19630473114181937	-2.3395845514910136	0.6560308759974934	1.040354072373259	2.3395845514910136	0.6560308759974934
**feature_6162**	ENSGALG00000013809	1.8822159515362682	-0.5256006797467602	0.6700033637042703	2.707980453089218	0.5256006797467602	0.6700033637042703
**feature_5686**	UNC5C	0.8083135988192562	-1.1259164951223974	0.6801182102654284	1.7747216528720298	1.1259164951223974	0.6801182102654284
**feature_6745**	PLPPR1	0.7159349018113412	-0.7917495510909667	0.6918575769432111	1.2468949543885381	0.7917495510909667	0.6918575769432111
**feature_5440**	CCSER1	1.47805915212664	-0.7085892446889526	0.6961193910636536	2.4172932858084546	0.7085892446889526	0.6961193910636536
**feature_5970**	FGFRL1	1.4318698036226825	-0.8337104706558472	0.7078904248191736	2.554987207151974	0.8337104706558472	0.7078904248191736
**feature_6144**	SETBP1	4.006925982718313	-0.5440732209586014	0.7078904248191736	5.829042670208994	0.5440732209586014	0.7078904248191736
**feature_9242**	ADGRL2	3.8914526114584196	-0.5993126692728785	0.7078904248191736	5.88259030628703	0.5993126692728785	0.7078904248191736
**feature_2490**	FAM171A1	0.8891449587011819	-0.617928347440226	0.7132069713714664	1.3692895511383334	0.617928347440226	0.7132069713714664
**feature_8571**	ZCCHC24	1.29330175811081	-0.7461166166097157	0.7132069713714664	2.172504092308864	0.7461166166097157	0.7132069713714664
**feature_4145**	AKT3	1.66281654614247	-0.5579978670801681	0.7381123478362688	2.447891934995903	0.5579978670801681	0.7381123478362688
**feature_1280**	ROBO1	8.290988056460371	-0.4886348797805353	0.7777896831844019	11.596888042043092	0.4886348797805353	0.7777896831844019
**feature_7689**	CCDC85C	1.0623550155910224	-0.5865641765308907	0.7777896831844019	1.5987794200441994	0.5865641765308907	0.7777896831844019
**feature_9295**	ZSWIM5	1.3279437694887781	-0.5534964748452273	0.7777896831844019	1.9506638856998604	0.5534964748452273	0.7777896831844019
**feature_5685**	BMPR1B	1.8822159515362682	-0.5969572856099535	0.7858766544186221	2.8456743744327375	0.5969572856099535	0.7858766544186221
**feature_3336**	TOX	1.0623550155910224	-0.6924976212799558	0.7860767605334921	1.7211740167939944	0.6924976212799558	0.7860767605334921
**feature_9132**	HMCN1	0.7274822389373307	-0.6688653126654512	0.7860767605334921	1.162748669123054	0.6688653126654512	0.7860767605334921
**feature_11962**	PREX1	1.2817544209848206	-0.504989355895944	0.7944539756223695	1.820619626653203	0.504989355895944	0.7944539756223695
**feature_12875**	SUMO2	0.92378697007915	-0.5554432443524312	0.8193516224132459	1.3616398888414711	0.5554432443524312	0.8193516224132459
**feature_14702**	CADM1	3.129328361143121	-0.5576343054821624	0.834048499396882	4.597447040414181	0.5576343054821624	0.834048499396882
**feature_5482**	STOX2	1.2240177353548738	-0.52175952498771	0.8369176844171785	1.7594223282783055	0.52175952498771	0.8369176844171785
**feature_3645**	PTK2	2.251730739567928	-0.4762477253630373	0.840867363209488	3.128711879416639	0.4762477253630373	0.840867363209488
**feature_676**	RFX4	3.383369777914887	-0.6275468934265251	0.8458595045752582	5.217069686460019	0.6275468934265251	0.8458595045752582
**feature_5362**	GAB1	0.7852189245672775	-0.5975737620862575	0.8542653161386191	1.1933473183105028	0.5975737620862575	0.8542653161386191
**feature_1956**	HMGB1	1.2471124096068524	-0.5012741403234451	0.8568062294285627	1.7670719905751677	0.5012741403234451	0.8568062294285627
**feature_4251**	MAP3K4	1.3279437694887781	-0.5307763983451439	0.8653220243332669	1.9200652365124116	0.5307763983451439	0.8653220243332669
**feature_875**	SCUBE1	1.1778283868509163	-0.6137201916878402	0.8670314602036451	1.8053203020594786	0.6137201916878402	0.8670314602036451
**feature_906**	LRP6	0.9815236557090968	-0.5087746713277144	0.8670314602036451	1.3998882003257822	0.5087746713277144	0.8670314602036451
**feature_6700**	FAM172A	1.570437849134555	-0.4783523136879605	0.8670314602036451	2.1878034169025886	0.4783523136879605	0.8670314602036451
**feature_240**	PTPRZ1	4.133946691104196	-0.5818489189015432	0.8675798579729549	6.1732774735677935	0.5818489189015432	0.8675798579729549
**feature_2656**	TBC1D5	1.8822159515362682	-0.446242888133476	0.8771379456796496	2.5626368694488364	0.446242888133476	0.8771379456796496
**feature_6552**	PTCH1	1.3394911066147674	-0.5690187469592793	0.8771379456796496	1.9889121971841714	0.5690187469592793	0.8771379456796496
**feature_14396**	PAX7	1.2240177353548738	-0.576901079180171	0.8771379456796496	1.8282692889500654	0.576901079180171	0.8771379456796496
**feature_14629**	KIRREL3	0.8083135988192562	-0.7374637814345152	0.8771379456796496	1.353990226544609	0.7374637814345152	0.8771379456796496
**feature_6361**	SREK1	0.7159349018113412	-0.542722003251052	0.8833572109633625	1.048003734670121	0.542722003251052	0.8833572109633625
**feature_2961**	FARS2	0.9584289814571181	-0.4867655972770366	0.8833664616363398	1.3463405642477468	0.4867655972770366	0.8833664616363398
**feature_3094**	ENSGALG00000013155	1.570437849134555	-0.6797676012580885	0.8833664616363398	2.516738895667663	0.6797676012580885	0.8833664616363398
**feature_13740**	HOXB3	0.831408273071235	-0.561502913977407	0.8847889739076006	1.2315956297948139	0.561502913977407	0.8847889739076006
**feature_3459**	RUNX1T1	0.6697455533073837	-0.8049588258851958	0.8894711595066443	1.1780479937167785	0.8049588258851958	0.8894711595066443
**feature_11254**	EBF1	1.0392603413390438	-0.9535313792246649	0.8897702564031665	2.0195108463716203	0.9535313792246649	0.8897702564031665
**feature_8034**	BMPR2	1.0969970269689906	-0.48472645200343817	0.9015142979679105	1.5375821216693017	0.48472645200343817	0.9015142979679105
**feature_2089**	FAT3	3.360275103662908	-0.49311593899715667	0.9060708875122785	4.719841637163976	0.49311593899715667	0.9060708875122785
**feature_3119**	ENSGALG00000013212	1.2471124096068524	-0.4950421866119305	0.9083286499484474	1.7594223282783055	0.4950421866119305	0.9083286499484474
**feature_3028**	FBXL7	1.3048490952367995	-0.5033999594138387	0.9179395177176818	1.851218275840652	0.5033999594138387	0.9179395177176818
**feature_11494**	LFNG	2.9099289557493226	-0.5106329366323608	0.9198768974172541	4.138467302602449	0.5106329366323608	0.9198768974172802
**feature_3863**	JAG1	0.5311775077955112	-1.1330130235693996	0.9221162072396963	1.1780479937167785	1.1330130235693996	0.9221162072396963
**feature_3840**	MEIS1	4.180136039608154	-0.448748347326573	0.9232922154804409	5.691348748865475	0.448748347326573	0.9232922154804409
**feature_2674**	TOP2B	0.6928402275593625	-0.5251342517683667	0.9249670911605847	1.0021057608889479	0.5251342517683667	0.9249670911605847
**feature_3184**	LDLRAD4	0.8545029473232137	-0.5489809937227343	0.9317525358158337	1.2545446166854004	0.5489809937227343	0.9317525358158469
**feature_6601**	ENSGALG00000000220	1.351038443740757	-0.45957175817384455	0.9317525358158337	1.858867938137514	0.45957175817384455	0.9317525358158469
**feature_8148**	ENSGALG00000031069	1.3279437694887781	-0.5076927852321027	0.9317622669509705	1.8894665873249628	0.5076927852321027	0.9317622669509705
**feature_10052**	RORA	2.517319493465684	-0.4408214315549437	0.9476169064907232	3.41174938440054	0.4408214315549437	0.9476169064907232
**feature_78**	PLXNA4	1.9861419856701725	-0.6668472137555201	0.9657840723343484	3.1516608663072256	0.6668472137555201	0.9657840723343484
**feature_5864**	NCAPG	0.9584289814571181	-0.4621035430427675	0.9768406088659126	1.3233915773571603	0.4621035430427675	0.9768406088659126
**feature_13462**	BCAS3	1.3394911066147674	-0.4659252539951759	0.9768406088659126	1.851218275840652	0.4659252539951759	0.9768406088659126
**feature_16677**	FUS	9.203227689413533	-0.36275488032118863	0.978049871639164	11.795779261761508	0.36275488032118863	0.978049871639164
**feature_4418**	NKAIN2	2.8406449329933863	-0.5372517011406273	0.9818221108469032	4.115518315711863	0.5372517011406273	0.9818221108469032
**feature_7283**	ORAOV1	1.1200917012209695	-0.454979108609386	0.9821749020263766	1.5375821216693017	0.454979108609386	0.9821749020263766
**feature_3150**	TSHZ1	1.0046183299610756	-0.4358616285997587	0.9855445039435514	1.3616398888414711	0.4358616285997587	0.9855445039435514
**feature_7115**	TEAD1	1.8706686144102789	-0.4550667385204822	0.98805652319032	2.5626368694488364	0.4550667385204822	0.98805652319032
**feature_8691**	PLCE1	1.073902352717012	-0.43431744633091063	0.9934564810954563	1.4534358364038176	0.43431744633091063	0.9934564810954563
**feature_3728**	XPO1	1.6050798605125232	-0.47695130745008085	0.9954916154392702	2.2337013906837617	0.47695130745008085	0.9954916154392702
**feature_3984**	CENPF	1.6859112203944489	-0.4785916658313366	0.9954916154392702	2.3484463251366945	0.4785916658313366	0.9954916154392702
**feature_158**	RELN	0.6581982161813944	-1.1384589755663836	0.9981672383024977	1.4610854987006798	1.1384589755663836	0.9981672383024977
**feature_2853**	EZH2	1.8706686144102789	-0.4114774699727993	0.9981672383024977	2.4861402464802143	0.4114774699727993	0.9981672383024977
**feature_3401**	ZFHX4	1.8360266030323107	-0.4293993779700619	0.9981672383024977	2.4708409218864897	0.4293993779700619	0.9981672383024977
**feature_4039**	PRKCE	1.0623550155910224	-0.44217426719571584	0.9981672383024977	1.4457861741069553	0.44217426719571584	0.9981672383024977
**feature_4061**	DAAM2	0.7967662616932669	-0.5950774083121885	0.9981672383024977	1.2086466429042273	0.5950774083121885	0.9981672383024977
**feature_6698**	SLF1	0.8083135988192562	-0.49054904096399843	0.9981672383024977	1.1397996822324674	0.49054904096399843	0.9981672383024977
**feature_7050**	NAV2	1.8937632886622575	-0.4201946870739085	0.9981672383024977	2.5320382202613874	0.4201946870739085	0.9981672383024977
**feature_7240**	PPP6R3	0.8083135988192562	-0.44163944048305176	0.9981672383024977	1.1015513707481566	0.44163944048305176	0.9981672383024977
**feature_9865**	RSRC1	0.8083135988192562	-0.4515549093481345	0.9981672383024977	1.1092010330450186	0.4515549093481345	0.9981672383024977
**feature_12199**	PPIL2	0.7736715874412882	-0.4635659811212536	0.9981672383024977	1.0709527215607078	0.4635659811212536	0.9981672383024977
**feature_12250**	NCOR2	1.2702070838588313	-0.43724056603788136	0.9981672383024977	1.7211740167939944	0.43724056603788136	0.9981672383024977
**feature_12837**	SPAG9	1.4549644778746613	-0.4562157847380838	0.9981672383024977	1.9965618594810337	0.4562157847380838	0.9981672383024977
**feature_13739**	HOXB4	1.1200917012209695	-0.4188194453139676	0.9981672383024977	1.4993338101849907	0.4188194453139676	0.9981672383024977
**feature_15081**	HNRNPR	1.073902352717012	-0.43431744633091063	0.9981672383024977	1.4534358364038176	0.43431744633091063	0.9981672383024977
**feature_76**	EXOC4	3.8683579372064405	-0.2188323920304034	1	4.49035176825811	0.2188323920304034	1
**feature_77**	CHCHD3	1.7782899174023639	-0.04224366030708038	1	1.8282692889500654	0.04224366030708038	1
**feature_93**	TAF3	1.3625857808667463	-0.23775889537204092	1	1.6064290823410614	0.23775889537204092	1
**feature_161**	KMT2E	1.2355650724808631	-0.17228980609337335	1	1.39223853802892	0.17228980609337335	1
**feature_174**	COG5	1.351038443740757	-0.028680307694163576	1	1.3769392134351957	0.028680307694163576	1
**feature_186**	SLC2A13	1.0969970269689906	-0.07444248269522899	1	1.155099006826192	0.07444248269522899	1
**feature_201**	TAFA5	2.898381618623333	-0.3350643544536316	1	3.6488889156032682	0.3350643544536316	1
**feature_203**	BRD1	2.9330236300013013	-0.064582459568862	1	3.059864918744879	0.064582459568862	1
**feature_221**	PLXNB2	1.3048490952367995	-0.4051359430989728	1	1.7288236790908567	0.4051359430989728	1
**feature_263**	ENSGALG00000053114	1.7782899174023639	-0.27490441709735547	1	2.1495551054182775	0.27490441709735547	1
**feature_271**	DOCK4	1.3279437694887781	-0.23999700202844576	1	1.5681807708567506	0.23999700202844576	1
**feature_300**	ARID2	1.6974585575204382	-0.229930100913734	1	1.9889121971841714	0.229930100913734	1
**feature_320**	SRGAP1	3.0138549898832268	-0.11614672805583948	1	3.2587561384632964	0.11614672805583948	1
**feature_335**	GRIP1	1.5819851862605443	-0.3418720024869326	1	2.0042115217778957	0.3418720024869326	1
**feature_346**	CPSF6	1.10854436409498	-0.02101799341462149	1	1.1245003576387431	0.02101799341462149	1
**feature_356**	CNOT2	2.1131626940560557	-0.006856586639294004	1	2.118956456230829	0.0068565866392940045	1
**feature_383**	SYT1	2.6443402018515667	-0.10514623013645563	1	2.8380247121358755	0.10514623013645563	1
**feature_385**	PPP1R12A	1.5819851862605443	-0.14215680129157438	1	1.7441230036845812	0.14215680129157438	1
**feature_578**	TNRC6B	1.0854496898430013	-0.10840840233675293	1	1.1703983314199162	0.10840840233675293	1
**feature_603**	DDX17	1.4434171407486718	-0.04152786022249366	1	1.4840344855912664	0.04152786022249366	1
**feature_714**	HIPK2	1.166281049724927	-0.3756767485449528	1	1.5146331347787152	0.3756767485449528	1
**feature_715**	KIAA1549	1.2471124096068524	-0.46984163248678806	1	1.7288236790908567	0.46984163248678806	1
**feature_752**	BICD1	1.2471124096068524	-0.04417258147805725	1	1.2851432658728492	0.04417258147805725	1
**feature_757**	DENND5B	1.3972277922447145	-0.28164003233021795	1	1.6982250299034078	0.28164003233021795	1
**feature_788**	CECR2	1.5357958377565868	-0.22803132887686806	1	1.7976706397626165	0.22803132887686806	1
**feature_822**	PDE3A	1.6397218718904913	-0.4659252539951756	1	2.2643000398712103	0.4659252539951756	1
**feature_832**	SOX5	5.450343123466985	-0.45799584860178966	1	7.466070401737505	0.45799584860178966	1
**feature_894**	ATXN10	1.016165667087065	-0.02320688025674107	1	1.0327044100763967	0.02320688025674107	1
**feature_918**	CCDC91	1.4549644778746613	-0.05214940328024331	1	1.506983472481853	0.05214940328024331	1
**feature_946**	TSPAN9	1.258659746732842	-0.11393353371239623	1	1.3616398888414711	0.11393353371239623	1
**feature_1105**	GSK3B	1.9630473114181937	-0.12290797203186798	1	2.134255780824553	0.12290797203186798	1
**feature_1129**	LSAMP	2.8290975958673967	-0.3175859050628975	1	3.518844656556611	0.3175859050628975	1
**feature_1203**	BBX	1.47805915212664	-0.09373717204887044	1	1.5758304331536128	0.09373717204887044	1
**feature_1251**	POU2F1	1.2702070838588313	-0.05168983125941834	1	1.315741915060298	0.05168983125941834	1
**feature_1281**	ROBO2	7.471127120515126	-0.26761476511668336	1	8.965404211922495	0.26761476511668336	1
**feature_1289**	NRIP1	4.3071567479940365	-0.26332670202335606	1	5.155872388085121	0.26332670202335606	1
**feature_1367**	HLCS	0.8775976215751925	-0.2739739462228644	1	1.0633030592638455	0.2739739462228644	1
**feature_1424**	ENSGALG00000016217	2.4826774820877158	-0.018533741544572324	1	2.5090892333370801	0.018533741544572324	1
**feature_1430**	CASK	2.6327928647255776	-0.006901909512580184	1	2.639133492417458	0.006901909512580184	1
**feature_1434**	DDX3X	1.1778283868509163	-0.010379161651829477	1	1.1856976560136407	0.010379161651829477	1

**feature_1436**	ENSGALG00000016236	1.3394911066147674	-0.04095863747260037	1	1.3769392134351957	0.04095863747260037	1
**feature_1457**	DMD	2.424940796457769	-0.21785528732601045	1	2.815075725245289	0.21785528732601045	1
**feature_1458**	IL1RAPL1	0.9353343072051394	-0.10920790085590533	1	1.0097554231858101	0.10920790085590533	1
**feature_1480**	CNKSR2	3.036949664135206	-0.180864541831383	1	3.4346983712911268	0.180864541831383	1
**feature_1533**	SHROOM2	1.1547337125989374	-0.2370817644852613	1	1.3616398888414711	0.2370817644852613	1
**feature_1578**	HERC2	1.143186375472948	-0.015548793641660258	1	1.155099006826192	0.015548793641660258	1
**feature_1619**	MAP4K4	2.332562099449854	-0.14702098906980754	1	2.5779361940425605	0.14702098906980754	1
**feature_1634**	NCK1	1.3279437694887781	-0.19012109538887634	1	1.5146331347787152	0.19012109538887634	1
**feature_1689**	ANKRD10	3.1639703725210886	-0.20300742486872103	1	3.6335895910095437	0.20300742486872103	1
**feature_1690**	ENSGALG00000031009	1.7436479060243957	-0.2350123190858382	1	2.050109495559069	0.2350123190858382	1
**feature_1750**	UBAC2	1.1547337125989374	-0.3078548388986421	1	1.430486849513231	0.3078548388986421	1
**feature_1757**	FARP1	2.2055413910639707	-0.3422148075356859	1	2.7921267383547024	0.3422148075356859	1
**feature_1778**	GPC5	1.1778283868509163	-0.18440856142687842	1	1.3386909019508846	0.18440856142687842	1
**feature_1817**	DACH1	7.655884514530956	-0.25318389682118037	1	9.095448470969153	0.25318389682118037	1
**feature_1833**	TDRD3	1.6281745347645018	-0.029914675245043654	1	1.659976718419097	0.029914675245043654	1
**feature_1849**	DGKH	1.6743638832684593	-0.4600576504178613	1	2.3025483513555214	0.4600576504178613	1
**feature_1855**	ENOX1	0.7505769131893094	-0.7334055648601617	1	1.2545446166854004	0.7334055648601617	1
**feature_1862**	GTF2F2	0.9353343072051394	-0.1307410624055466	1	1.0250547477795344	0.1307410624055466	1
**feature_1870**	ZC3H13	1.0854496898430013	-0.25101279777955315	1	1.2927929281697115	0.25101279777955315	1
**feature_1918**	LHFPL6	1.6050798605125232	-0.10237341544302081	1	1.7211740167939944	0.10237341544302081	1
**feature_1938**	DCLK1	1.6859112203944489	-0.06978612026400721	1	1.7670719905751677	0.06978612026400721	1
**feature_1939**	NBEA	2.090068019804077	-0.30028761095596157	1	2.5702865317456984	0.30028761095596157	1
**feature_1945**	PDS5B	0.9815236557090968	-0.3161295933853185	1	1.2239459674979516	0.3161295933853185	1
**feature_1965**	PAN3	1.073902352717012	-0.046713177175179144	1	1.1092010330450186	0.046713177175179144	1
**feature_1992**	MICU2	1.1316390383469588	-0.23333196172551868	1	1.3310412396540223	0.23333196172551868	1
**feature_2009**	PSPC1	1.9745946485441832	-0.11449573221566789	1	2.134255780824553	0.11449573221566789	1
**feature_2019**	DDX10	1.1316390383469588	-0.1040489447805521	1	1.2162963052010893	0.1040489447805521	1
**feature_2084**	ENSGALG00000040433	0.8198609359452457	-0.4014238053088764	1	1.086252046154432	0.4014238053088764	1
**feature_2124**	TENM4	4.1454940282301855	-0.22112056862567364	1	4.819287247023184	0.22112056862567364	1
**feature_2354**	CTDSPL	0.8198609359452457	-0.43137702741050415	1	1.1092010330450186	0.43137702741050415	1
**feature_2373**	ACVR2B	2.898381618623333	-0.2921785416004552	1	3.5417936434471975	0.2921785416004552	1
**feature_2382**	KMT2C	1.9745946485441832	-0.007580528299155897	1	1.9812625348873092	0.0075805282991558975	1
**feature_2400**	PTPRN2	5.288680403703133	-0.1764574403086021	1	5.959086929255652	0.1764574403086021	1
**feature_2405**	ZMYND11	0.8891449587011819	-0.3644799904356302	1	1.1474493445293297	0.3644799904356302	1
**feature_2420**	PARD3	4.734408221655643	-0.07055755429025501	1	4.956981168366704	0.07055755429025501	1
**feature_2422**	ITGB1	0.9699763185831075	-0.10011861679562446	1	1.040354072373259	0.10011861679562446	1
**feature_2423**	EPC1	1.3625857808667463	-0.10162220738601196	1	1.4610854987006798	0.10162220738601196	1
**feature_2426**	ZEB1	1.0046183299610756	-0.27397394622286453	1	1.2162963052010893	0.27397394622286453	1
**feature_2434**	WAC	1.0854496898430013	-0.09900970433450336	1	1.162748669123054	0.09900970433450336	1
**feature_2436**	MPP7	1.016165667087065	-0.3620087937084995	1	1.3080922527634358	0.3620087937084995	1
**feature_2456**	ARHGAP21	2.009236659922151	-0.20819387158405633	1	2.317847675949246	0.20819387158405633	1
**feature_2464**	DNAJC1	1.5357958377565868	-0.2462550874957754	1	1.820619626653203	0.2462550874957754	1
**feature_2465**	MLLT10	2.332562099449854	-0.28917337304286117	1	2.8456743744327375	0.28917337304286117	1
**feature_2558**	PHF14	2.517319493465684	-0.20654601870257383	1	2.899222010510773	0.20654601870257383	1
**feature_2608**	IGF2BP3	3.452653800670823	-0.18828332867079678	1	3.9242767582903073	0.18828332867079678	1
**feature_2638**	JAZF1	0.7736715874412882	-0.400830225773291	1	1.0250547477795344	0.400830225773291	1
**feature_2783**	ARPP21	0.7736715874412882	-0.3792970642236497	1	1.0097554231858101	0.3792970642236497	1
**feature_2805**	BBS9	1.0623550155910224	-0.2731980951469523	1	1.2851432658728492	0.2731980951469523	1
**feature_2834**	GLI3	6.039257316892443	-0.12120586254784893	1	6.548110926114041	0.12120586254784893	1
**feature_2847**	TPK1	2.863739607245365	-0.18956779752520858	1	3.2587561384632964	0.18956779752520858	1
**feature_2876**	RECK	0.9815236557090968	-0.10415378799420891	1	1.0556533969669832	0.10415378799420891	1
**feature_2889**	ATP9B	1.1778283868509163	-0.1679204386383095	1	1.3233915773571603	0.1679204386383095	1
**feature_2909**	KIF13A	1.2240177353548738	-0.14574391453805036	1	1.353990226544609	0.14574391453805036	1
**feature_2981**	GMDS	2.806002921615418	-0.12819855886483356	1	3.059864918744879	0.12819855886483356	1
**feature_2998**	PHLPP1	0.9006922958271713	-0.24724807419448996	1	1.0709527215607078	0.24724807419448996	1
**feature_3024**	BASP1	1.570437849134555	-0.22033598262079113	1	1.8282692889500654	0.22033598262079113	1
**feature_3029**	ANKH	1.8937632886622575	-0.19490237523166576	1	2.164854430012002	0.19490237523166576	1
**feature_3034**	ENSGALG00000050676	2.2055413910639707	-0.016339528131660508	1	2.2260517283868997	0.016339528131660508	1
**feature_3074**	GRB10	1.7090058946464275	-0.13482345484987768	1	1.8741672627312385	0.13482345484987768	1
**feature_3128**	TEX10	1.200923061102895	-0.08093624478072886	1	1.269843941279125	0.08093624478072886	1
**feature_3152**	ZNF407	3.0715916755131736	-0.2785595003675876	1	3.717735876275028	0.2785595003675876	1
**feature_3170**	DOK6	1.016165667087065	-0.02320688025674107	1	1.0327044100763967	0.02320688025674107	1
**feature_3205**	PPP4R1	1.0623550155910224	-0.052227748879702426	1	1.1015513707481566	0.052227748879702426	1
**feature_3270**	CDH2	2.7251715617334926	-0.05411568159085234	1	2.822725387542151	0.05411568159085234	1
**feature_3293**	SPIDR	1.2355650724808631	-0.19574877891736225	1	1.4151875249195065	0.19574877891736225	1
**feature_3343**	NKAIN3	1.385680455118725	-0.33810428364258016	1	1.7517726659814432	0.33810428364258016	1
**feature_3352**	PDE7A	1.016165667087065	-0.3107834703533263	1	1.2621942789822627	0.3107834703533263	1
**feature_3368**	ARFGEF1	1.166281049724927	-0.13136955078006413	1	1.277493603575987	0.13136955078006413	1
**feature_3389**	STAU2	1.0046183299610756	-0.1017929708401842	1	1.0786023838575698	0.1017929708401842	1
**feature_3519**	UBR5	1.10854436409498	-0.04996936711075087	1	1.1474493445293297	0.04996936711075087	1
**feature_3554**	OXR1	1.6050798605125232	-0.28219445302783297	1	1.9506638856998604	0.28219445302783297	1
**feature_3571**	TRPS1	1.7436479060243957	-0.4303364972241155	1	2.3484463251366945	0.4303364972241155	1
**feature_3577**	EXT1	4.572745501891792	-0.033510842648571515	1	4.666294001085941	0.033510842648571515	1
**feature_3644**	RPLP1	1.1316390383469588	-0.3595496779289384	1	1.4534358364038176	0.3595496779289384	1
**feature_3664**	ZC3H3	0.9930709928350863	-0.2353205148189725	1	1.1703983314199162	0.2353205148189725	1
**feature_3727**	USP34	1.7090058946464275	-0.3164302613398253	1	2.126606118527691	0.3164302613398253	1
**feature_3733**	SPTBN1	1.4203224664966931	-0.08663777966553388	1	1.506983472481853	0.08663777966553388	1
**feature_3789**	ENSGALG00000037200	1.2471124096068524	-0.1190265761622708	1	1.353990226544609	0.1190265761622708	1
**feature_3790**	KIF16B	1.1778283868509163	-0.02875769096668404	1	1.200996980607365	0.02875769096668404	1
**feature_3802**	LCLAT1	0.831408273071235	-0.3405939838162897	1	1.0556533969669832	0.3405939838162897	1
**feature_3818**	FBXO11	1.5357958377565868	-0.16556724193021466	1	1.7211740167939944	0.16556724193021466	1
**feature_3853**	CDC42BPA	1.4318698036226825	-0.35858252154067405	1	1.8359189512469274	0.35858252154067405	1
**feature_3980**	ESRRG	5.7621212258686985	-0.14092147680646283	1	6.333920381801899	0.14092147680646283	1
**feature_4023**	MTA3	0.7967662616932669	-0.3696572942781725	1	1.0327044100763967	0.3696572942781725	1
**feature_4053**	BRE	1.0969970269689906	-0.026187314088007646	1	1.1168506953418809	0.026187314088007646	1
**feature_4070**	BTBD9	0.8775976215751925	-0.34436327411426226	1	1.1168506953418809	0.34436327411426226	1
**feature_4071**	ZFAND3	1.5242485006305975	-0.1506109008217158	1	1.6905753676065456	0.1506109008217158	1
**feature_4106**	BIRC6	2.0438786713001194	-0.1931318816990535	1	2.3331470005429704	0.1931318816990535	1
**feature_4143**	ADSS	0.7967662616932669	-0.3482836434856056	1	1.0174050854826724	0.3482836434856056	1
**feature_4176**	ARID4B	1.3972277922447145	-0.11009675248952289	1	1.506983472481853	0.11009675248952289	1
**feature_4186**	PCNX2	0.9122396329531607	-0.3087178955475986	1	1.1321500199356052	0.3087178955475986	1
**feature_4233**	AFDN	2.2979200880718857	-0.32986370441914725	1	2.8839226859170486	0.32986370441914725	1
**feature_4247**	QKI	5.369511763585059	-0.09418578520615606	1	5.714297735756062	0.09418578520615606	1
**feature_4280**	SASH1	1.6974585575204382	-0.26811329480162216	1	2.042459833262207	0.26811329480162216	1
**feature_4293**	PLEKHG1	1.66281654614247	-0.33485618836988695	1	2.096007469340242	0.33485618836988695	1
**feature_4301**	ENSGALG00000013505	0.831408273071235	-0.3815242478711716	1	1.086252046154432	0.3815242478711716	1
**feature_4350**	NHSL1	2.1708993796860026	-0.3249684767181247	1	2.7156301153860802	0.3249684767181247	1
**feature_4369**	HBS1L	0.8660502844492032	-0.2615820394897407	1	1.040354072373259	0.2615820394897407	1
**feature_4459**	HDAC2	1.2124703982288845	-0.30560883486597673	1	1.4993338101849907	0.30560883486597673	1
**feature_4468**	FYN	1.258659746732842	-0.26674237699178865	1	1.5146331347787152	0.26674237699178865	1
**feature_4498**	SEC63	1.2471124096068524	-0.13514624152554777	1	1.3692895511383334	0.13514624152554777	1
**feature_4502**	PDSS2	1.050807678465033	-0.14530335660843852	1	1.162748669123054	0.14530335660843852	1
**feature_4514**	LIN28B	1.4318698036226825	-0.25947437652774963	1	1.7135243544971324	0.25947437652774963	1
**feature_4528**	PNISR	3.418011789292855	-0.10104019490821381	1	3.6565385779001307	0.10104019490821381	1
**feature_4566**	ZNF292	1.0277130042130544	-0.2142451225347517	1	1.1933473183105028	0.2142451225347517	1
**feature_4600**	SENP6	1.0623550155910224	-0.12970650155741992	1	1.162748669123054	0.12970650155741992	1
**feature_4608**	EEF1A1	1.5127011635046081	-0.1744382726719501	1	1.70587469220027	0.1744382726719501	1
**feature_4627**	ADGRB3	0.9930709928350863	-0.2446583793983087	1	1.1780479937167785	0.2446583793983087	1
**feature_4647**	DST	1.200923061102895	-0.4720190119078502	1	1.667626380715959	0.4720190119078502	1
**feature_4856**	MSRA	0.92378697007915	-0.4025443452027917	1	1.2239459674979516	0.4025443452027917	1
**feature_4915**	CDC5L	0.8660502844492032	-0.24036555358004486	1	1.0250547477795344	0.24036555358004486	1
**feature_5040**	OGT	1.385680455118725	-0.07004306073395361	1	1.4534358364038176	0.07004306073395361	1
**feature_5095**	RBMXL1	1.351038443740757	-0.17988611178988678	1	1.5299324593724395	0.17988611178988678	1
**feature_5117**	CHM	1.10854436409498	-0.001389186665688962	1	1.1092010330450186	0.0013891866656889629	1
**feature_5119**	ZNF711	0.7852189245672775	-0.43223603013959677	1	1.0633030592638455	0.43223603013959677	1
**feature_5190**	ATRX	3.1524230353950995	-0.1807241267219346	1	3.564742630337784	0.1807241267219346	1
**feature_5225**	THOC2	1.9514999742922043	-0.06278246552265176	1	2.0348101709653448	0.06278246552265176	1
**feature_5266**	MAMLD1	1.1200917012209695	-0.3203295819146041	1	1.3998882003257822	0.3203295819146041	1
**feature_5271**	AFF2	3.175517709647078	-0.04441999959577725	1	3.2664058007601584	0.04441999959577725	1
**feature_5280**	TMEM131L	0.831408273071235	-0.39157791253509455	1	1.0939017084512943	0.39157791253509455	1
**feature_5308**	RAPGEF2	1.9976893227961618	-0.33485618836988684	1	2.516738895667663	0.33485618836988684	1
**feature_5323**	SPOCK3	1.200923061102895	-0.362567869491853	1	1.545231783966164	0.362567869491853	1
**feature_5370**	ZNF827	1.0969970269689906	-0.38875739347271593	1	1.4381365118100933	0.38875739347271593	1
**feature_5467**	TET2	1.2471124096068524	-0.15899298347991547	1	1.39223853802892	0.15899298347991547	1
**feature_5547**	HNRNPDL	1.3972277922447145	-0.019898943517944523	1	1.4151875249195065	0.019898943517944523	1
**feature_5560**	WDFY3	1.7321005688984064	-0.03725887530200078	1	1.7747216528720298	0.03725887530200078	1
**feature_5606**	ANKRD17	1.258659746732842	-0.1458175947321822	1	1.39223853802892	0.1458175947321822	1
**feature_5619**	YTHDC1	1.258659746732842	-0.08954937508543709	1	1.3386909019508846	0.08954937508543709	1
**feature_5624**	FAT4	1.5588905120085657	-0.4838859655008494	1	2.180153754605726	0.4838859655008494	1

**feature_5663**	ANK2	1.2124703982288845	-0.5290470663539755	1	1.7517726659814432	0.5290470663539755	1
**feature_5688**	TSPAN5	2.251730739567928	-0.23555947843585234	1	2.6467831547143206	0.23555947843585234	1
**feature_5714**	CENPE	1.5473431748825763	-0.035101591063060766	1	1.5834800954504749	0.035101591063060766	1
**feature_5766**	SCFD2	0.8660502844492032	-0.34345904654414827	1	1.1015513707481566	0.34345904654414827	1
**feature_5775**	FRYL	1.1893757239769056	-0.14566597946843196	1	1.315741915060298	0.14566597946843196	1
**feature_5865**	LCORL	0.7505769131893094	-0.44389894766517696	1	1.0250547477795344	0.44389894766517696	1
**feature_5916**	AFAP1	1.0623550155910224	-0.20323553697938507	1	1.2239459674979516	0.20323553697938507	1
**feature_5934**	HTT	0.831408273071235	-0.3301773678709513	1	1.048003734670121	0.3301773678709513	1
**feature_5955**	ENSGALG00000050742	1.8591212772842893	-0.00826540609074522	1	1.8665176004343762	0.00826540609074522	1
**feature_6034**	C20orf194	1.2817544209848206	-0.31836806558131797	1	1.5987794200441994	0.31836806558131797	1
**feature_6035**	ATRN	1.3394911066147674	-0.1849689401641197	1	1.5222827970755775	0.1849689401641197	1
**feature_6050**	EXOC6B	0.9353343072051394	-0.16244992213288473	1	1.048003734670121	0.16244992213288473	1
**feature_6080**	NOP56	1.385680455118725	-0.3063954239152421	1	1.7135243544971324	0.3063954239152421	1
**feature_6101**	ENSGALG00000033770	1.10854436409498	-0.29538040504992835	1	1.3616398888414711	0.29538040504992835	1
**feature_6263**	ENSGALG00000003605	0.8660502844492032	-0.3234212933076033	1	1.086252046154432	0.3234212933076033	1
**feature_6309**	15,00 ARL	1.1316390383469588	-0.2167490775299145	1	1.315741915060298	0.2167490775299145	1
**feature_6351**	CWC27	0.9353343072051394	-0.15195754831524239	1	1.040354072373259	0.15195754831524239	1
**feature_6418**	TNPO1	1.5935325233865338	-0.05408762969377112	1	1.6523270561222347	0.05408762969377112	1
**feature_6438**	RFX3	1.200923061102895	-0.3338565728503256	1	1.5146331347787152	0.3338565728503256	1
**feature_6454**	UHRF2	1.073902352717012	-0.2319280496420867	1	1.2621942789822627	0.2319280496420867	1
**feature_6457**	PTPRD	1.9861419856701725	-0.1716060967583226	1	2.2337013906837617	0.1716060967583226	1
**feature_6473**	CCDC171	1.2702070838588313	-0.28948694559418925	1	1.552881446263026	0.28948694559418925	1
**feature_6475**	BNC2	2.03233133417413	-0.39095514609945425	1	2.6620824793080446	0.39095514609945425	1
**feature_6550**	AOPEP	0.9006922958271713	-0.28760581181063083	1	1.1015513707481566	0.28760581181063083	1
**feature_6581**	AUH	0.8545029473232137	-0.311941796421885	1	1.0633030592638455	0.311941796421885	1
**feature_6612**	FBXL17	1.755195243150385	-0.0428328761784067	1	1.8053203020594786	0.0428328761784067	1
**feature_6675**	ZNF462	1.6397218718904913	-0.3284217302452409	1	2.0577591578559313	0.3284217302452409	1
**feature_6676**	RAD23B	1.3279437694887781	-0.3083839770086959	1	1.6446773938253725	0.3083839770086959	1
**feature_6699**	KIAA0825	0.8891449587011819	-0.2345997078887202	1	1.048003734670121	0.2345997078887202	1
**feature_6746**	PALM2AKAP2	1.5127011635046081	-0.20628713871129	1	1.7441230036845812	0.20628713871129	1
**feature_6759**	ECPAS	0.8775976215751925	-0.19997336477908767	1	1.0097554231858101	0.19997336477908767	1
**feature_6911**	ZCCHC7	1.2240177353548738	-0.07940141970935422	1	1.2927929281697115	0.07940141970935422	1
**feature_7071**	KIF18A	1.016165667087065	-0.257672133893764	1	1.2162963052010893	0.257672133893764	1
**feature_7078**	MPPED2	1.016165667087065	-0.012559636057232561	1	1.0250547477795344	0.012559636057232561	1
**feature_7120**	GALNT18	2.1362573683080344	-0.34805208983116587	1	2.7156301153860802	0.34805208983116587	1
**feature_7121**	EIF4G2	1.050807678465033	-0.07774007279580376	1	1.1092010330450186	0.07774007279580376	1
**feature_7128**	SBF2	2.090068019804077	-0.2345997078887203	1	2.4555415972927657	0.2345997078887203	1
**feature_7140**	DENND2B	1.3972277922447145	-0.20183932111709824	1	1.6064290823410614	0.20183932111709824	1
**feature_7223**	RPLP2	0.8660502844492032	-0.27207441330738297	1	1.048003734670121	0.27207441330738297	1
**feature_7282**	CCND1	2.2632780766939176	-0.003744771317188755	1	2.2643000398712103	0.0037447713171887553	1
**feature_7368**	PHF21A	1.9514999742922043	-0.04100552312740002	1	2.0042115217778957	0.04100552312740002	1
**feature_7433**	NUMB	0.9930709928350863	-0.10808532652286457	1	1.0709527215607078	0.10808532652286457	1
**feature_7453**	SRSF5	1.351038443740757	-0.15819104069056789	1	1.506983472481853	0.15819104069056789	1
**feature_7479**	GPHN	2.0785206826780875	-0.11620272713879753	1	2.2490007152774862	0.11620272713879753	1
**feature_7501**	DPH6	1.1200917012209695	-0.272501056823989	1	1.353990226544609	0.272501056823989	1
**feature_7511**	G2E3	2.03233133417413	-0.00996111034757452	1	2.042459833262207	0.00996111034757452	1
**feature_7514**	STRN3	2.1131626940560557	-0.0875878587521508	1	2.2413510529806238	0.0875878587521508	1
**feature_7522**	NPAS3	11.674357834375257	-0.36512183428106004	1	14.985688439553046	0.36512183428106004	1
**feature_7539**	RALGAPA1	1.1200917012209695	-0.272501056823989	1	1.353990226544609	0.272501056823989	1
**feature_7546**	MIPOL1	0.8891449587011819	-0.25535826805551753	1	1.0633030592638455	0.25535826805551753	1
**feature_7631**	FOXN3	1.5127011635046081	-0.3212796610012213	1	1.8894665873249628	0.3212796610012213	1
**feature_7673**	DICER1	1.1200917012209695	-0.04469513930117683	1	1.155099006826192	0.04469513930117683	1
**feature_7683**	PAPOLB	0.9584289814571181	-0.14844306413900554	1	1.0633030592638455	0.14844306413900554	1
**feature_7693**	EML1	2.066973345552098	-0.16755188708671032	1	2.317847675949246	0.16755188708671032	1
**feature_7704**	PPP2R5C	1.29330175811081	-0.050730126194909075	1	1.3386909019508846	0.050730126194909075	1
**feature_7714**	RCOR1	0.8545029473232137	-0.311941796421885	1	1.0633030592638455	0.311941796421885	1
**feature_7724**	CKB	0.8660502844492032	-0.41147746997279955	1	1.155099006826192	0.41147746997279955	1
**feature_7753**	BRF1	1.2124703982288845	-0.04110902002768402	1	1.2468949543885381	0.04110902002768402	1
**feature_7780**	MNAT1	0.8775976215751925	-0.2427230122384247	1	1.040354072373259	0.2427230122384247	1
**feature_7917**	SPAG16	0.9006922958271713	-0.1951595630460359	1	1.0327044100763967	0.1951595630460359	1
**feature_7956**	HDAC4	1.6281745347645018	-0.1382472970331171	1	1.7900209774657543	0.1382472970331171	1
**feature_7974**	PCNT	1.6166271976385125	-0.2662795544328639	1	1.9430142234029981	0.2662795544328639	1
**feature_8061**	ABI2	0.9815236557090968	-0.3071408101580638	1	1.2162963052010893	0.3071408101580638	1
**feature_8098**	HNRNPA3	2.3210147623238644	-0.052474005608283525	1	2.40199396121473	0.052474005608283525	1
**feature_8124**	PPP1R9B	1.0046183299610756	-0.2918958542201269	1	1.2315956297948139	0.2918958542201269	1
**feature_8189**	RBMS1	2.4480354707097476	-0.051807281093886104	1	2.5320382202613874	0.051807281093886104	1
**feature_8257**	BIN1	1.0046183299610756	-0.17121437179766363	1	1.1321500199356052	0.17121437179766363	1
**feature_8263**	PTPN4	0.9815236557090968	-0.22361745459578053	1	1.1474493445293297	0.22361745459578053	1
**feature_8339**	DARS	0.7736715874412882	-0.4635659811212536	1	1.0709527215607078	0.4635659811212536	1
**feature_8352**	GTDC1	1.166281049724927	-0.35376197211651217	1	1.4916841478881286	0.35376197211651217	1
**feature_8389**	PKP4	1.2471124096068524	-0.11089869527887053	1	1.3463405642477468	0.11089869527887053	1
**feature_8393**	BAZ2B	4.237872725238101	-0.057840515358098976	1	4.398555820695764	0.057840515358098976	1
**feature_8418**	BMPR1A	1.5242485006305975	-0.14409759386304144	1	1.6829257053096836	0.14409759386304144	1
**feature_8472**	JMJD1C	2.5057721563396944	-0.18245195630390443	1	2.8380247121358755	0.18245195630390443	1
**feature_8482**	ANK3	4.053115331222271	-0.11191718811389162	1	4.367957171508315	0.11191718811389162	1
**feature_8523**	ENSGALG00000031604	1.4203224664966931	-0.034709987621672767	1	1.4534358364038176	0.034709987621672767	1
**feature_8573**	ZMIZ1	6.1316360139003585	-0.1731786577269907	1	6.89234572947284	0.1731786577269907	1
**feature_8577**	KCNMA1	0.9584289814571181	-0.5814024714151117	1	1.4381365118100933	0.5814024714151117	1
**feature_8578**	LRMDA	1.47805915212664	-0.49300735546939767	1	2.080708144746518	0.49300735546939767	1
**feature_8619**	GBF1	1.2471124096068524	-0.018332576542797685	1	1.2621942789822627	0.018332576542797685	1
**feature_8779**	SUFU	1.050807678465033	-0.006856586639294046	1	1.0556533969669832	0.006856586639294046	1
**feature_8798**	SH3PXD2A	1.5011538263786188	-0.3724079052942072	1	1.9430142234029981	0.3724079052942072	1
**feature_8876**	FGFR2	1.47805915212664	-0.10760934663042787	1	1.5911297577473371	0.10760934663042787	1
**feature_8918**	DOCK1	2.4826774820877158	-0.17228980609337352	1	2.7921267383547024	0.17228980609337352	1
**feature_8924**	ENSGALG00000025996	0.9815236557090968	-0.24260055796335336	1	1.162748669123054	0.24260055796335336	1
**feature_8926**	EBF3	1.1778283868509163	-0.0648269456742058	1	1.2315956297948139	0.0648269456742058	1
**feature_8935**	INPP5A	0.9930709928350863	-0.3172621283551486	1	1.239245292091676	0.3172621283551486	1
**feature_8982**	ASPM	1.9976893227961618	-0.35655125946920574	1	2.554987207151974	0.35655125946920574	1
**feature_8987**	CDC73	0.8198609359452457	-0.29683490403167667	1	1.0097554231858101	0.29683490403167667	1
**feature_9013**	MTA1	1.47805915212664	-0.2582312096771173	1	1.7670719905751677	0.2582312096771173	1
**feature_9052**	PBX1	2.03233133417413	-0.3404767010716603	1	2.5702865317456984	0.3404767010716603	1
**feature_9096**	COP1	1.016165667087065	-0.08549115851108363	1	1.0786023838575698	0.08549115851108363	1
**feature_9130**	TPR	1.1547337125989374	-0.20447709905333372	1	1.3310412396540223	0.20447709905333372	1
**feature_9166**	PTBP2	1.0046183299610756	-0.09159720373429571	1	1.0709527215607078	0.09159720373429571	1
**feature_9179**	GCLM	1.0277130042130544	-0.04890739058809104	1	1.0633030592638455	0.04890739058809104	1
**feature_9180**	FNBP1L	1.5011538263786188	-0.2909438046653108	1	1.8359189512469274	0.2909438046653108	1
**feature_9188**	EVI5	0.9584289814571181	-0.3045622660562875	1	1.1856976560136407	0.3045622660562875	1
**feature_9200**	ZNF644	0.8660502844492032	-0.28249102925272135	1	1.0556533969669832	0.28249102925272135	1
**feature_9202**	ZNF326	0.9353343072051394	-0.38484234346933266	1	1.2239459674979516	0.38484234346933266	1
**feature_9248**	FUBP1	1.7436479060243957	-0.33347495797152615	1	2.1954530791994507	0.33347495797152615	1
**feature_9256**	ST6GALNAC3	1.4549644778746613	-0.3885770678627206	1	1.9047659119186873	0.3885770678627206	1
**feature_9269**	PTPRF	2.6443402018515667	-0.3341717438053504	1	3.327603099135056	0.3341717438053504	1
**feature_9281**	ENSGALG00000045795	0.7621242503152987	-0.453912736293196	1	1.048003734670121	0.453912736293196	1
**feature_9338**	ELAVL4	1.755195243150385	-0.4983855351959644	1	2.478490584183352	0.4983855351959644	1
**feature_9381**	SSBP3	1.0854496898430013	-0.021530951656785854	1	1.1015513707481566	0.021530951656785854	1
**feature_9395**	DAB1	0.7043875646853519	-0.5016752789443777	1	1.0021057608889479	0.5016752789443777	1
**feature_9446**	SRSF11	1.2355650724808631	-0.1078010796416598	1	1.3310412396540223	0.1078010796416598	1
**feature_9458**	SLC44A5	0.9930709928350863	-0.012928093482524576	1	1.0021057608889479	0.012928093482524576	1
**feature_9460**	RABGGTB	1.385680455118725	-0.27397394622286464	1	1.6752760430128213	0.27397394622286464	1
**feature_9461**	MSH4	1.0277130042130544	-0.23250732892748	1	1.2086466429042273	0.23250732892748	1
**feature_9501**	HS6ST1	1.8360266030323107	-0.318368065581318	1	2.2872490267617973	0.318368065581318	1
**feature_9539**	TRA2B	1.0854496898430013	-0.27625008927857625	1	1.315741915060298	0.27625008927857625	1
**feature_9598**	ENSGALG00000028415	0.92378697007915	-0.6107862781962061	1	1.4151875249195065	0.6107862781962061	1
**feature_9611**	SPSB4	0.9006922958271713	-0.22663779451920388	1	1.0556533969669832	0.22663779451920388	1
**feature_9646**	TRIP12	1.5127011635046081	-0.21257340155872118	1	1.7517726659814432	0.21257340155872118	1
**feature_9661**	U2SURP	1.4665118150006506	-0.15967031956225963	1	1.6370277315285102	0.15967031956225963	1
**feature_9733**	NCL	1.351038443740757	-0.052396376667971034	1	1.3998882003257822	0.052396376667971034	1
**feature_9735**	ENSGALG00000043329	1.351038443740757	-0.34901338302614593	1	1.7211740167939944	0.34901338302614593	1
**feature_9782**	EIF4A2	1.4896064892526293	-0.3374768885290227	1	1.8818169250281007	0.3374768885290227	1
**feature_9808**	TBL1XR1	1.2817544209848206	-0.1039684349984008	1	1.3769392134351957	0.1039684349984008	1
**feature_9817**	FNDC3B	1.4087751293707038	-0.02355181631119685	1	1.430486849513231	0.02355181631119685	1
**feature_9835**	MECOM	1.9284053000402257	-0.4327879762182114	1	2.600885180933147	0.4327879762182114	1
**feature_9854**	SMC4	1.3625857808667463	-0.31755960658516064	1	1.6982250299034078	0.31755960658516064	1
**feature_10063**	MPHOSPH10	1.016165667087065	-0.03377612196692836	1	1.040354072373259	0.03377612196692836	1
**feature_10073**	THSD4	0.9353343072051394	-0.3018529787983013	1	1.155099006826192	0.3018529787983013	1
**feature_10089**	TCF12	3.51039048630077	-0.05059574074086082	1	3.6259399287126817	0.05059574074086082	1
**feature_10090**	ENSGALG00000037723	1.3279437694887781	-0.02110202492822494	1	1.3463405642477468	0.02110202492822494	1
**feature_10095**	PRTG	7.805999897168817	-0.14673724657002385	1	8.613519746266835	0.14673724657002385	1
**feature_10215**	CHD2	1.5819851862605443	-0.20984360880314878	1	1.8282692889500654	0.20984360880314878	1

**feature_10249**	IGDCC3	2.678982213229535	-0.36107251653555616	1	3.4346983712911268	0.36107251653555616	1
**feature_10346**	PBRM1	0.9584289814571181	-0.15871139959283262	1	1.0709527215607078	0.15871139959283262	1
**feature_10435**	CENPP	1.050807678465033	-0.2716198063898388	1	1.269843941279125	0.2716198063898388	1
**feature_10447**	VGLL4	1.200923061102895	-0.10662288844437834	1	1.2927929281697115	0.10662288844437834	1
**feature_10465**	IQSEC1	0.9699763185831075	-0.4778199648014955	1	1.353990226544609	0.4778199648014955	1
**feature_10484**	CACNA1D	1.0277130042130544	-0.2771763782612083	1	1.2468949543885381	0.2771763782612083	1
**feature_10593**	PTPRG	2.852192270119376	-0.1749575826523202	1	3.212858164682123	0.1749575826523202	1
**feature_10606**	LRIG1	3.6143165204346746	-0.09413664458108872	1	3.8477801353216856	0.09413664458108872	1
**feature_10621**	FOXP1	2.5404141677176626	-0.11542800691104271	1	2.746228764573529	0.11542800691104271	1
**feature_10631**	CHL1	1.200923061102895	-0.5552129474342489	1	1.7670719905751677	0.5552129474342489	1
**feature_10663**	TMCC1	1.1200917012209695	-0.07289203113182885	1	1.1780479937167785	0.07289203113182885	1
**feature_10756**	CNOT1	1.1200917012209695	-0.18908504863635145	1	1.277493603575987	0.18908504863635145	1
**feature_10811**	SLC12A4	1.1778283868509163	-0.001101348063818702	1	1.1780479937167785	0.0011013480638187023	1
**feature_10822**	CHD9	1.8129319287803318	-0.13006453482415092	1	1.9812625348873092	0.13006453482415092	1
**feature_10823**	TOX3	1.2124703982288845	-0.20711897146661323	1	1.3998882003257822	0.20711897146661323	1
**feature_10827**	NKD1	2.6905295503555244	-0.09570160206703227	1	2.868623361323324	0.09570160206703227	1
**feature_10860**	TSHZ3	0.9930709928350863	-0.04534957117490199	1	1.0250547477795344	0.04534957117490199	1
**feature_10921**	WWOX	3.360275103662908	-0.11343661431109231	1	3.6259399287126817	0.11343661431109231	1
**feature_10989**	ANKRD11	2.6443402018515667	-0.209843608803149	1	3.052215256448017	0.209843608803149	1
**feature_11025**	ZFHX3	0.7736715874412882	-0.3792970642236497	1	1.0097554231858101	0.3792970642236497	1
**feature_11093**	ANKHD1	1.3625857808667463	-0.10911674393293619	1	1.4687351609975419	0.10911674393293619	1
**feature_11129**	MATR3	2.77136091023745	-0.029969594259150023	1	2.822725387542151	0.029969594259150023	1
**feature_11150**	SLIT3	4.237872725238101	-0.5299672068437635	1	6.104430512896034	0.5299672068437635	1
**feature_11332**	HNRNPAB	1.7898372545283532	-0.28594658788894045	1	2.180153754605726	0.28594658788894045	1
**feature_11513**	ACTB	0.92378697007915	-0.24155246853048698	1	1.0939017084512943	0.24155246853048698	1
**feature_11613**	RBBP6	1.29330175811081	-0.13045731866564308	1	1.4151875249195065	0.13045731866564308	1
**feature_11696**	RBFOX1	1.200923061102895	-0.18908504863635145	1	1.3692895511383334	0.18908504863635145	1
**feature_11733**	CREBBP	1.1893757239769056	-0.02392070711606259	1	1.2086466429042273	0.02392070711606259	1
**feature_11865**	RBM39	2.990760315631248	-0.10678608001074097	1	3.212858164682123	0.10678608001074097	1
**feature_11959**	ZMYND8	1.10854436409498	-0.1691166324037555	1	1.2468949543885381	0.1691166324037555	1
**feature_11960**	NCOA3	1.1200917012209695	-0.16308984010340694	1	1.2545446166854004	0.16308984010340694	1
**feature_11979**	CDH4	1.9168579629142362	-0.06669216679104939	1	2.0042115217778957	0.06669216679104939	1
**feature_11999**	DIDO1	1.5588905120085657	-0.07890213088592857	1	1.6446773938253725	0.07890213088592857	1
**feature_12061**	TPX2	0.9584289814571181	-0.06355416655249263	1	1.0021057608889479	0.06355416655249263	1
**feature_12124**	GNAS	1.10854436409498	-0.20388205056443207	1	1.277493603575987	0.20388205056443207	1
**feature_12191**	HIC2	0.9930709928350863	-0.3694134104062553	1	1.2851432658728492	0.3694134104062553	1
**feature_12213**	ARVCF	1.5357958377565868	-0.03887523701435214	1	1.5758304331536128	0.03887523701435214	1
**feature_12320**	ATXN2	1.073902352717012	-0.24059291076921419	1	1.269843941279125	0.24059291076921419	1
**feature_12384**	ZNRF3	1.2124703982288845	-0.260985843445349	1	1.4534358364038176	0.260985843445349	1
**feature_12484**	MSI1	1.166281049724927	-0.060980222888666225	1	1.2162963052010893	0.060980222888666225	1
**feature_12564**	MED13L	1.8822159515362682	-0.3622644954941231	1	2.4172932858084546	0.3622644954941231	1
**feature_12605**	MYH10	1.5011538263786188	-0.1594141819600089	1	1.6752760430128213	0.1594141819600089	1
**feature_12644**	SRSF2	0.8198609359452457	-0.4114774699727993	1	1.0939017084512943	0.4114774699727993	1
**feature_12673**	H3F3B	0.7621242503152987	-0.43285112076536636	1	1.0327044100763967	0.43285112076536636	1
**feature_12721**	ZNF207	1.166281049724927	-0.27821093910933525	1	1.4151875249195065	0.27821093910933525	1
**feature_12841**	LUC7L3	2.182446716811992	-0.09900970433450351	1	2.3331470005429704	0.09900970433450351	1
**feature_12937**	ZNF618	1.9745946485441832	-0.056675077827325726	1	2.050109495559069	0.056675077827325726	1
**feature_12949**	RPL12	1.050807678465033	-0.20984360880314895	1	1.2162963052010893	0.20984360880314895	1
**feature_12981**	PAPPA	3.360275103662908	-0.2742209618969358	1	4.054321017336965	0.2742209618969358	1
**feature_13181**	PBX3	1.5357958377565868	-0.2995845899019022	1	1.8894665873249628	0.2995845899019022	1
**feature_13184**	LMX1B	0.92378697007915	-0.2111788194869682	1	1.0709527215607078	0.2111788194869682	1
**feature_13250**	AUTS2	15.115464297920091	-0.05259986228121427	1	15.620610410192608	0.05259986228121427	1
**feature_13281**	CUX1	3.267896406654993	-0.14437339982995856	1	3.602990941822095	0.14437339982995856	1
**feature_13483**	SRSF1	0.8775976215751925	-0.41147746997279944	1	1.1703983314199162	0.41147746997279944	1
**feature_13877**	BRCA1	1.7205532317724168	-0.18828332867079667	1	1.9583135479967226	0.18828332867079667	1
**feature_14300**	SKI	3.787526577324515	-0.1422908371574103	1	4.169065951789897	0.1422908371574103	1
**feature_14309**	GNB1	1.2471124096068524	-0.22825564591702913	1	1.4610854987006798	0.22825564591702913	1
**feature_14432**	PRDM2	0.9930709928350863	-0.29945085223868806	1	1.2239459674979516	0.29945085223868806	1
**feature_14565**	ENSGALG00000038064	0.9353343072051394	-0.375853560242078	1	1.2162963052010893	0.375853560242078	1
**feature_14692**	ENSGALG00000006818	1.016165667087065	-0.257672133893764	1	1.2162963052010893	0.257672133893764	1
**feature_15057**	PUM1	1.8129319287803318	-0.061893032182571805	1	1.8894665873249628	0.061893032182571805	1
**feature_15134**	SRRM1	0.92378697007915	-0.20091048403314113	1	1.0633030592638455	0.20091048403314113	1
**feature_15197**	SFPQ	2.1362573683080344	-0.022065778369450006	1	2.164854430012002	0.022065778369450006	1
**feature_15390**	SRSF3	1.5242485006305975	-0.1506109008217158	1	1.6905753676065456	0.1506109008217158	1
**feature_15418**	NUCKS1	1.47805915212664	-0.05846169730133999	1	1.5375821216693017	0.05846169730133999	1
**feature_15501**	RPL10A	0.7736715874412882	-0.47376174822714207	1	1.0786023838575698	0.47376174822714207	1
**feature_15766**	HNRNPM	1.2124703982288845	-0.34889293259477666	1	1.545231783966164	0.34889293259477666	1
**feature_16038**	PTPRS	2.1708993796860026	-0.12535945102596435	1	2.363745649730419	0.12535945102596435	1
**feature_16295**	ILF2	1.6974585575204382	-0.04122789244079361	1	1.7441230036845812	0.04122789244079361	1
