# Supplementary material for: RIPOR2 promotes multinucleation of melanoma cells downstream of the RAS/ERK oncogenic pathway
Source: iScience. 2026 Apr 15;29(5):115734. doi: 10.1016/j.isci.2026.115734 (PMC13145898; doi:10.1016/j.isci.2026.115734)
Supplement: Table S2. List of biopsies analyzed with the anti-RIPOR2 antibody [file mmc3.pdf]

Table S2: List of biopsies analyzed with the anti-RIPOR2 antibody

Lesion description	RIPOR2 expressed in melanocytes
Benign congenital melanocytic nevus, BRAF^{V600E}	YES
Benign melanocytic nevus, BRAF^{V600E}	YES
Benign melanocytic nevus ,with multinucleated cells and neuroid maturation, BRAF^{V600E}	YES
Benign melanocytic nevus, with multinucleated cells, BRAF^{V600E}	YES
Early melanoma, BRAF^{V600E}	YES
Early melanoma	YES
Early melanoma, BRAF$_{V600E}$	NO
Advanced melanoma	YES
Advanced melanoma BRAF^{V600E}	NO
